# Intercellular Transfer of CD155 Mediated by Melanoma Extracellular Vesicles Downregulates DNAM-1 Expression on NK Cells

**DOI:** 10.3390/ijms27198699

**Published:** 2026-09-29

**Authors:** Li-Ying Wu, Luize Goncalves Lima, Sunyoung Ham, Mina Lim, Su-Ho Park, Yue Su, Xuanxuan Li, Edna Pei Zhi Chai, Matthias Braun, Yong-Soo Choi, Tobias Bald, Andreas Möller

**Affiliations:** 1 Tumour Microenvironment Laboratory, QIMR Berghofer Medical Research Institute, Herston, QLD 4006, Australia; abigail.lyw128@gmail.com (L.-Y.W.); peizhi.edna@gmail.com (E.P.Z.C.); 2 School of Biomedical Sciences, Faculty of Health, Queensland University of Technology, Brisbane, QLD 4001, Australia; 3 JC STEM Lab of Personalised Cancer Medicine, Li Ka Shing Institute of Health Sciences, The Chinese University of Hong Kong, Hong Kong SAR, China; sunyoungham@cuhk.edu.hk (S.H.); su-ho.park@cuhk.edu.hk (S.-H.P.); yuesu@link.cuhk.edu.hk (Y.S.); 1155123725@link.cuhk.edu.hk (X.L.); 4 Department of Otorhinolaryngology, Faculty of Medicine, The Chinese University of Hong Kong, Shatin, Hong Kong SAR, China; 5 Department of Bioconvergence Science, Graduate School, CHA University, Seongnam 13488, Republic of Korea; nimmina@naver.com; 6 Department of Paediatric Haematology, Oncology and Immunodeficiency, Justus-Liebig-University Giessen, 35392 Giessen, Germany; matthias.braun@paediat.med.uni-giessen.de; 7 Department of Medicinal Biosciences and Bioengineering, Bioengineering, Inha University, Incheon 22212, Republic of Korea; yschoi93@inha.ac.kr; 8 Department of Biological Sciences and Bioengineering, Inha University, Incheon 22212, Republic of Korea; 9 Institute for Experimental Oncology, University Hospital Bonn, 53127 Bonn, Germany; tobias.bald@ukbonn.de

**Keywords:** melanoma, CD155, extracellular vesicles, natural killer (NK) cells, DNAM-1 (CD226), tumour microenvironment

## Abstract

Melanoma is a highly immunogenic cancer, and immunotherapy is the first-line treatment for patients with advanced disease. However, CD155 overexpression in melanoma has been associated with resistance to immunotherapy. Whether CD155 can be transferred from tumour cells via extracellular vesicles (EVs), and how this transfer affects immune responses in melanoma, remains unclear. To investigate melanoma cell transfer of CD155 in vivo, B16F10-WT and B16F10-CD155KO melanoma cells were subcutaneously injected into CD155^−/−^ mice. We show that CD155 is transferred from melanoma cells to several immune cell populations infiltrating the tumour, at least partly through EV secretion and uptake. DNAM-1 expression was significantly reduced on natural killer (NK) cells in B16F10-WT tumours compared with B16F10-CD155KO tumours. We also found that CD155 was enclosed and enriched in different melanoma EV subtypes. Finally, incubation of NK cells with CD155^+^ melanoma EVs increased cellular CD155 levels and led to downregulation of DNAM-1. In summary, melanoma-derived CD155 is enriched in melanoma EVs and transferred to immune cells, especially NK cells. Moreover, cancer-derived CD155^+^ EVs increase CD155 expression on NK cells and are associated with reduced DNAM-1 expression.

## 1. Introduction

Melanoma is one of the most commonly diagnosed cancers in young adults [1] and has the highest mutation rate among all cancer types [2]. It is also the leading cause of death from skin cancer. Because melanoma is highly immunogenic [3,4], immunotherapy is commonly used to treat patients with advanced disease [3,5]. Combination treatment with immune checkpoint inhibitors, such as nivolumab and ipilimumab, significantly improves outcomes compared with monotherapy [6,7,8]. However, some patients fail to respond to immunotherapy or eventually develop resistance [9,10]. Melanoma cells can acquire resistance through multiple mechanisms, including the induction of natural killer (NK) cell dysfunction [5,11,12].

Poliovirus receptor (PVR), also known as CD155, regulates both activating and inhibitory immune signalling by interacting with several receptors, including DNAX accessory molecule-1 (DNAM-1; also known as CD226), T cell immunoreceptor with Ig and ITIM domains (TIGIT), and CD96, on NK cells and CD8 T cells [13,14,15]. Under physiological conditions, CD155 expression is very low, but it can be induced during inflammation and in cancer [5,13,16]. DNAM-1 ligation can cooperate with LFA-1 signalling to promote cytokine secretion and cytotoxicity in NK cells and CD8^+^ T cells. In NK cells, phosphorylation of the DNAM-1 cytoplasmic ITT-like motif by Src-family kinases enables Grb2 recruitment and activation of Vav1, PI3K, and PLCγ1, with downstream ERK and AKT activation, calcium flux, granule polarization, cytokine production, and cytotoxicity [17,18,19]. AKT-mediated phosphorylation of FOXO1 has been proposed to support NK cell activation by promoting FOXO1 nuclear exclusion and inactivation [5,20,21]. Conversely, CD155 ligation of TIGIT results in phosphorylation of its ITT-like motif and recruitment of Grb2 and SHIP-1, which suppresses PI3K–AKT and MAPK signalling and limits NK cell activation and cytotoxicity [22,23]. We previously showed that high CD155 expression in melanoma cells from pretreatment tumour samples is associated with resistance to immune checkpoint blockade [24,25]. CD155 overexpression can exert multiple immunosuppressive effects [5]. It can bind TIGIT expressed on tumour-infiltrating CD8 T and NK cells, reducing cytotoxicity and contributing to an immunosuppressive tumour microenvironment [11,26]. In addition, CD155 can induce internalization and proteasomal degradation of the activating receptor DNAM-1 in CD8 T and NK cells [24,27,28]. Overall, CD155 is an important mediator of immune suppression in melanoma, and understanding its function may aid the development of new therapies.

Extracellular vesicles (EVs) play critical roles in cell–cell communication and in regulating cell phenotype and behaviour [29]. EVs comprise heterogeneous populations that are often operationally classified into apoptotic bodies, large EVs (lEVs), and small EVs (sEVs) based on size, density, and biogenesis [30,31]. lEVs, including microvesicles, are typically released by outward budding of the plasma membrane [30,31]. Exosomes belong to the sEV population and are nanosized particles with diameters of 30–150 nm that are typically generated within endosomal compartments and released upon fusion of multivesicular bodies with the plasma membrane. After the secretion into the extracellular space, lEVs and sEVs can then be taken up by many cell types [30,32,33]. Because EV cargo is specific to the cell of origin, its protein, lipid, and nucleic acid content partly reflects the phenotype of the secreting cells [34]. Cancer-derived EVs contribute to tumour progression and drug resistance [35,36,37,38,39,40], promote tumour growth [41], and modulate anti-tumour immune responses [35,42]. Therefore, understanding how tumour cells communicate with and reprogram immune cells through EVs may inform the development of new therapies.

Within the tumour microenvironment, activation of CD8 T and NK cells is essential for anti-tumour immune responses [11,24]. CD155 overexpression has been reported in multiple cancer types [24,43,44,45] and is associated with dysfunction of both CD8 T and NK cells. CD155^+^ sEVs have been detected in serum samples obtained from patients with liver cancer and are associated with NK cell dysfunction [27]. In addition, CD155^+^ sEVs isolated from disseminated cancer cells of a melanoma patient were shown to suppress cytokine secretion by CD8 T cells [27,46]. Therefore, in this study, we assessed whether CD155 from melanoma cells can be packaged into extracellular vesicles, transferred to immune cells, and potentially suppress anti-melanoma immune responses within the tumour microenvironment.

## 2. Results

### 2.1. Melanoma-Derived CD155 Is Transferred to Tumour-Infiltrating Immune Cells

**Figure 1:**
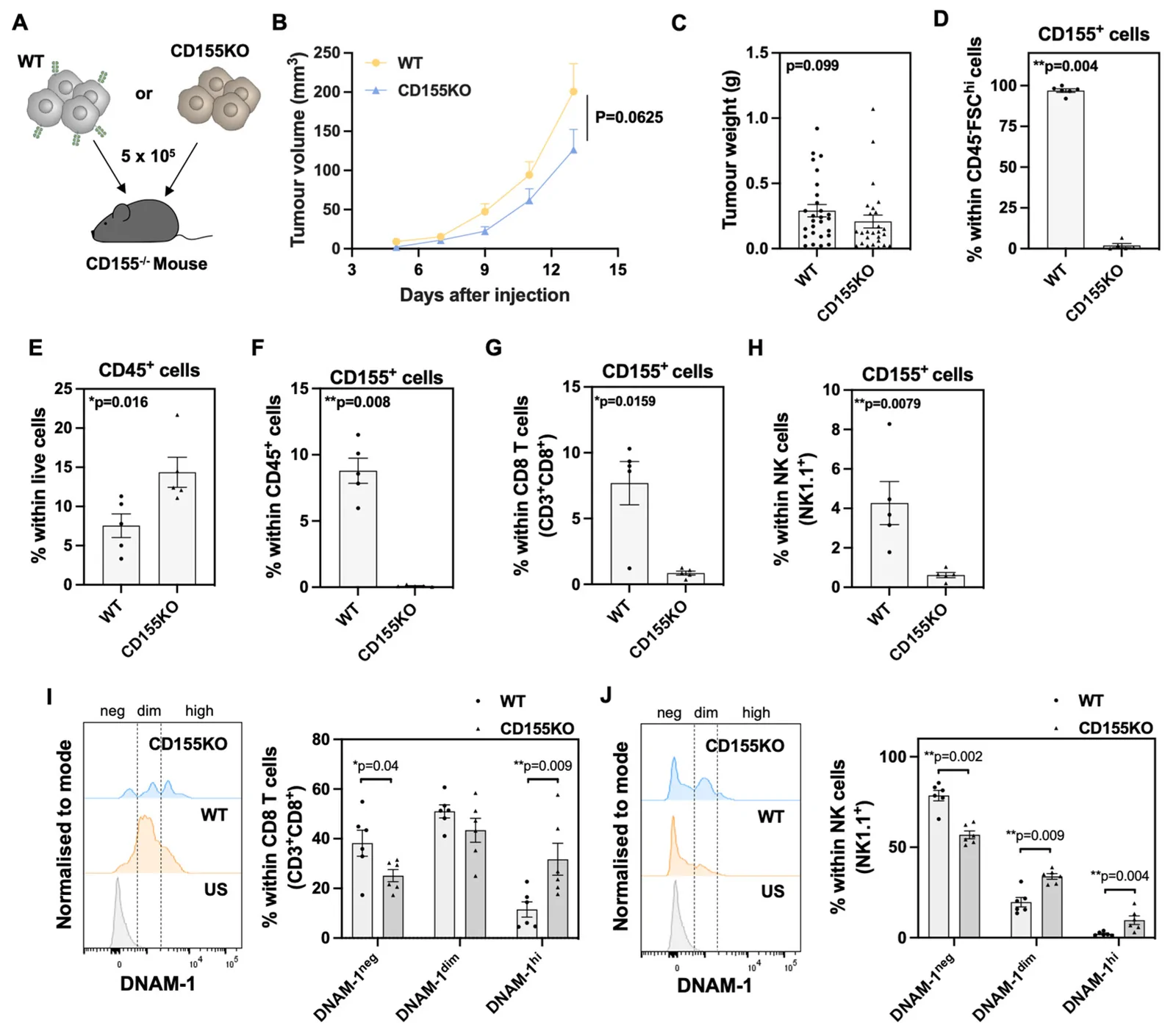
CD155 is transferred from melanoma cells to immune cells infiltrating B16F10-WT tumours. (**A**) Schematic of the B16F10 mouse primary tumour model. Briefly, 5 × 10^5^ B16F10-WT or B16F10-CD155KO cells were subcutaneously injected into CD155^−/−^ mice. (**B**) Tumour growth curves for each group. Values are shown as mean tumour volume at each time point. Error bars represent SEM (WT, n = 27 mice; CD155KO, n = 26 mice; cumulative data from five independent experiments). *p* values were analysed by the Wilcoxon test. (**C**) Individual tumours were dissected on day 14 post-injection. Individual tumour weights for each group are shown with mean and SEM (WT, n = 27 mice; CD155KO, n = 26 mice; cumulative data from five independent experiments). (**D**) Frequency of CD155^+^ cells within melanoma cell populations from WT and CD155KO tumours (n = 5 mice per group). (**E**) Frequency of CD45^+^ immune cells among live cells from WT and CD155KO tumours (n = 5 mice per group). (**F**) Frequency of CD155^+^ cells within CD45^+^ immune cells from WT and CD155KO tumours (n = 5 mice per group). (**G**) Frequency of CD155^+^ cells within CD8 T cells (CD3^+^CD8^+^) from WT and CD155KO tumours (n = 5 mice per group). (**H**) Frequency of CD155^+^ cells within NK cells (NK1.1^+^) from WT and CD155KO tumours (n = 5 mice per group). (**I**) Representative histograms (**left**) and quantified frequency (**right**) of DNAM-1^+^ cells within CD8 T cells (n = 6 mice per group). (**J**) Representative histograms (**left**) and quantified frequency (**right**) of DNAM-1^+^ cells within NK cells (n = 6 mice per group). Data are presented as mean ± SEM. * *p* < 0.05 and ** *p* <0.01, as analysed by the two-tailed Mann–Whitney U-test.

Since CD155 plays an important role in immune suppression in melanoma, we used B16F10 melanoma cells to investigate the contribution of tumour cell-derived CD155 to immunoregulation within the tumour microenvironment. We first confirmed that CD155 was deleted in B16F10-CD155 knockout (CD155KO) cells (Figure S1A,B). To assess the impact of melanoma cells on immune responses in vivo, B16F10 wild-type (WT) or B16F10-CD155KO cells were injected subcutaneously into CD155^−/−^ mice to establish tumours (Figure 1A). Loss of CD155 in melanoma cells slightly reduced tumour growth compared with B16F10-WT cells in CD155^−/−^ mice (Figure 1B,C). CD155 expression was confirmed in melanoma cells (CD45^−^FSC^hi^; see Figure S2 for gating strategy) from B16F10-WT tumours but was absent in melanoma cells from B16F10-CD155KO tumours (Figure 1D). Consistent with the slightly reduced tumour growth, flow cytometric analysis revealed a significant increase in the frequency of CD45^+^ cells infiltrating CD155KO tumours compared with WT tumours (Figure 1E; see Figure S2 for gating strategy).

Interestingly, a significant fraction of immune cells infiltrating WT tumours in CD155^−/−^ mice were positive for surface CD155. Tumour-derived CD155 was detected on most immune cell populations, including macrophages (CD11b^+^F4/80^+^), dendritic cells (DCs; CD11c^+^MHCII^+^), monocytic and granulocytic myeloid cells (CD11b^+^Ly6C^+^Ly6G^−^, CD11b^+^Ly6C^+^Ly6G^+^ and CD11b^+^Ly6C^+^Ly6G^hi^), B cells (CD19^+^) (Figure S3), CD8 (CD3^+^CD8^+^) T cells and NK cells (NK1.1^+^) (Figure 1G,H) but not on CD4 (CD3^+^CD4^+^) T cells (Figure S3; see Figure S2 for gating strategy). Further analysis showed no significant difference in the composition of immune cell subsets infiltrating CD155KO and WT tumours (Figure S4). Notably, the frequencies of immune cell populations, particularly NK cells, correlated with tumour size (Figure S5). The proportion of NK cells was higher in smaller tumours (Figure S5B), although this was independent of CD155 expression.

The expression of the CD155 receptors DNAM-1 and TIGIT, which are key determinants of CD8^+^ T cell and NK cell function, was next evaluated on tumour-infiltrating immune cells [5]. DNAM-1, but not TIGIT, was significantly downregulated on both CD8^+^ T cells and NK cells infiltrating WT tumours compared with CD155KO tumours (Figure 1I,J and Figure S6). This effect was especially pronounced in NK cells, where we observed a marked reduction in DNAM-1^+^ cells, driven by decreases in both DNAM-1^dim^ and DNAM-1^hi^ populations and accompanied by an increase in DNAM-1^neg^ cells in WT tumours (Figure 1J). CD155-mediated internalization has been reported for both TIGIT and DNAM-1, with TIGIT proposed to be transiently internalized and recycled back to the cell surface, whereas DNAM-1 is preferentially routed for degradation [11,24]. These distinct trafficking and signalling mechanisms may contribute to the different DNAM-1 and TIGIT expression patterns observed in our model.

### 2.2. CD155-Dependent Effects Are Confined Largely to the Tumour Microenvironment

To investigate how CD155 expression in melanoma cells affects the systemic immune landscape, we assessed the frequencies of several immune cell subsets in the spleen, tumour-draining lymph nodes, and contralateral lymph nodes of WT- and CD155KO melanoma-bearing mice (Figures S7 and S8, respectively). Overall, only minor changes in immune cell frequencies were observed, suggesting that this effect was restricted to the tumour microenvironment. Consistent with this interpretation, the frequencies of DNAM-1–positive CD8^+^ T cells and NK cells in the spleens of tumour-bearing mice were similar between the WT and CD155KO groups (Figure S9). TIGIT expression was also examined on CD8^+^ T and NK cells in the tumour microenvironment and spleens of melanoma-bearing mice. In contrast to DNAM-1, TIGIT expression was not altered in tumour-infiltrating immune cells (Figure S6) but was significantly higher in CD8^+^ T and NK cells from the spleens of CD155KO- compared with WT tumour-bearing mice (Figure S9B,D). Overall, these data support the notion that CD155 is transferred from WT CD155^+^ melanoma cells to infiltrating immune cells and contributes to immunoregulation by downregulating the activating immune receptor DNAM-1 within the tumour microenvironment.

### 2.3. CD155 Is Enriched in Melanoma-Derived EVs and Transferred to NK Cells

**Figure 2:**
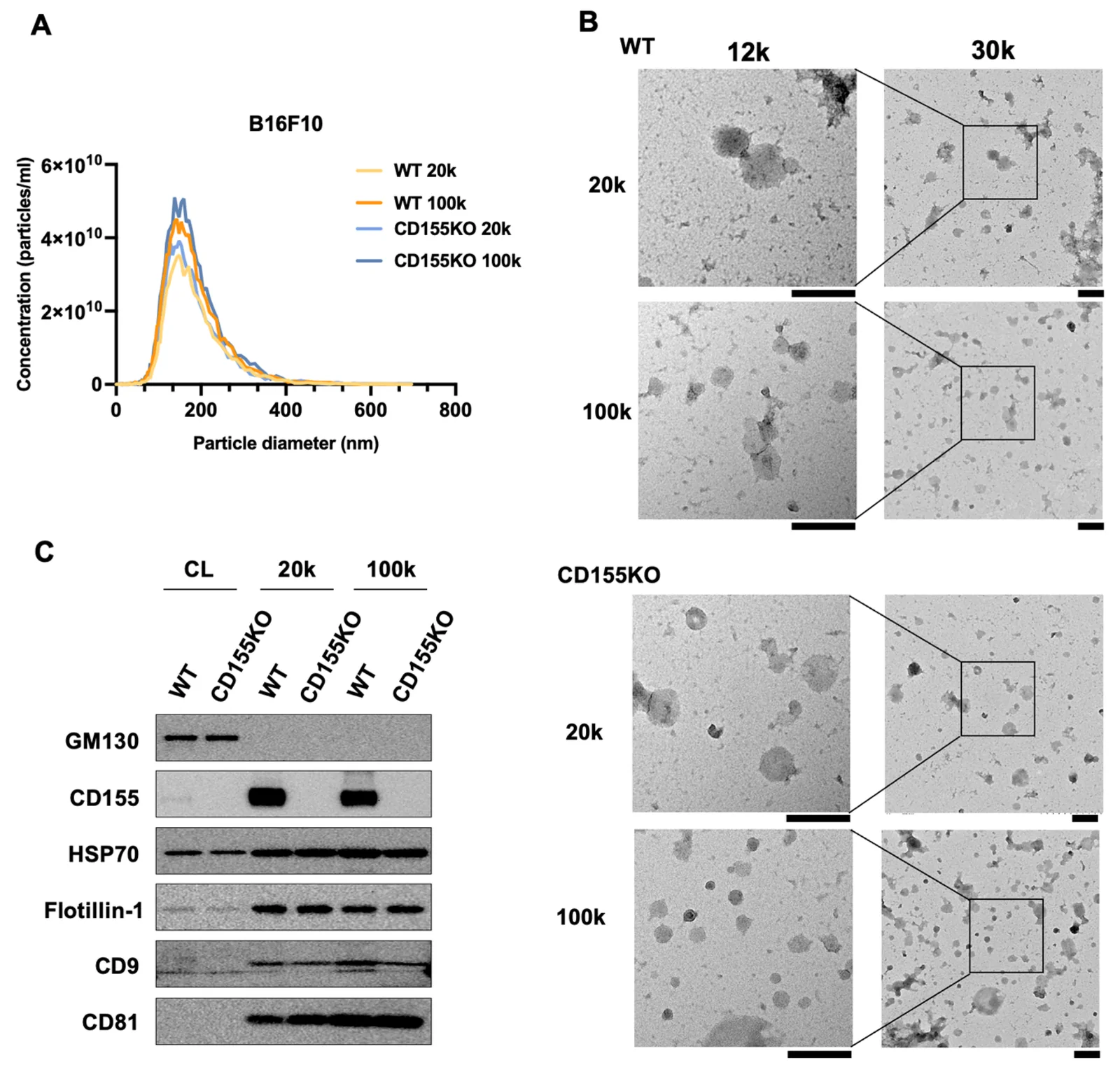
CD155 is enclosed within both 20k and 100k B16F10-derived EV subsets. (**A**) Size distribution of B16F10-WT- and B16F10-CD155KO-derived 20k and 100k EVs. (**B**) The morphology was analysed by TEM. Representative images were acquired at 12,000× and 30,000× magnification using an accelerating voltage of 100 kV and a beam current of 10 μA (scale bar, 200 nm). (**C**) Representative immunoblots of EV markers (CD81, 25 kDa; CD9, 25 kDa; flotillin-1, 48 kDa; HSP70, 70 kDa), the cell marker (cis-Golgi protein GM130, 112 kDa), and CD155 (100 kDa for mouse CD155) in cell lysates (CL) and 20k and 100k EVs isolated from conditioned media of B16F10-WT and B16F10-CD155KO cells. Equal amounts of protein (5 µg) were loaded for each sample to assess protein expression. Similar results were obtained from at least three biologically independent cell preparations. Source data are provided as a Source Data file.

Given these in vivo findings, we next examined whether CD155 transfer from melanoma cells to immune cells was mediated by EVs. Unlike in human samples, soluble CD155 was not detected in mouse models [47,48], suggesting that CD155 may instead be packaged within EVs and taken up by immune cells. To test this possibility, we isolated EVs from B16F10 WT and B16F10 CD155KO cells. Previous studies have shown that EVs isolated at different ultracentrifugation forces exhibit distinct protein expression profiles [49,50,51]. Therefore, we analysed EVs isolated from cancer cell supernatants at 20,000 *g* and 100,000 *g*. Conditioned medium was collected after 48 h of cell culture and first centrifuged at 500 *g* to remove cells and debris, and then the supernatant was centrifuged at 20,000 *g* to obtain 20k EV pellets (20k EVs). The remaining supernatant was further ultracentrifuged at 100,000 *g* to obtain 100k EV pellets (100k EVs). The size distributions of 20k and 100k EVs isolated from B16F10 WT and B16F10 CD155KO cells largely overlapped (Figure 2A). Transmission electron microscopy revealed spherical, membrane-bound vesicles consistent with the expected morphology of EVs (Figure 2B). EV markers, including flotillin-1, CD9, and CD81, were detected in both 20k and 100k EVs from B16F10 WT and CD155KO cells (Figure 2C). CD155 was present in WT EVs, with slightly lower levels in 100k than in 20k EVs (Figure 2C). Collectively, these data indicate that CD155 is enclosed within both 20k and 100k EV subsets derived from B16F10 melanoma cells.

**Figure 3:**
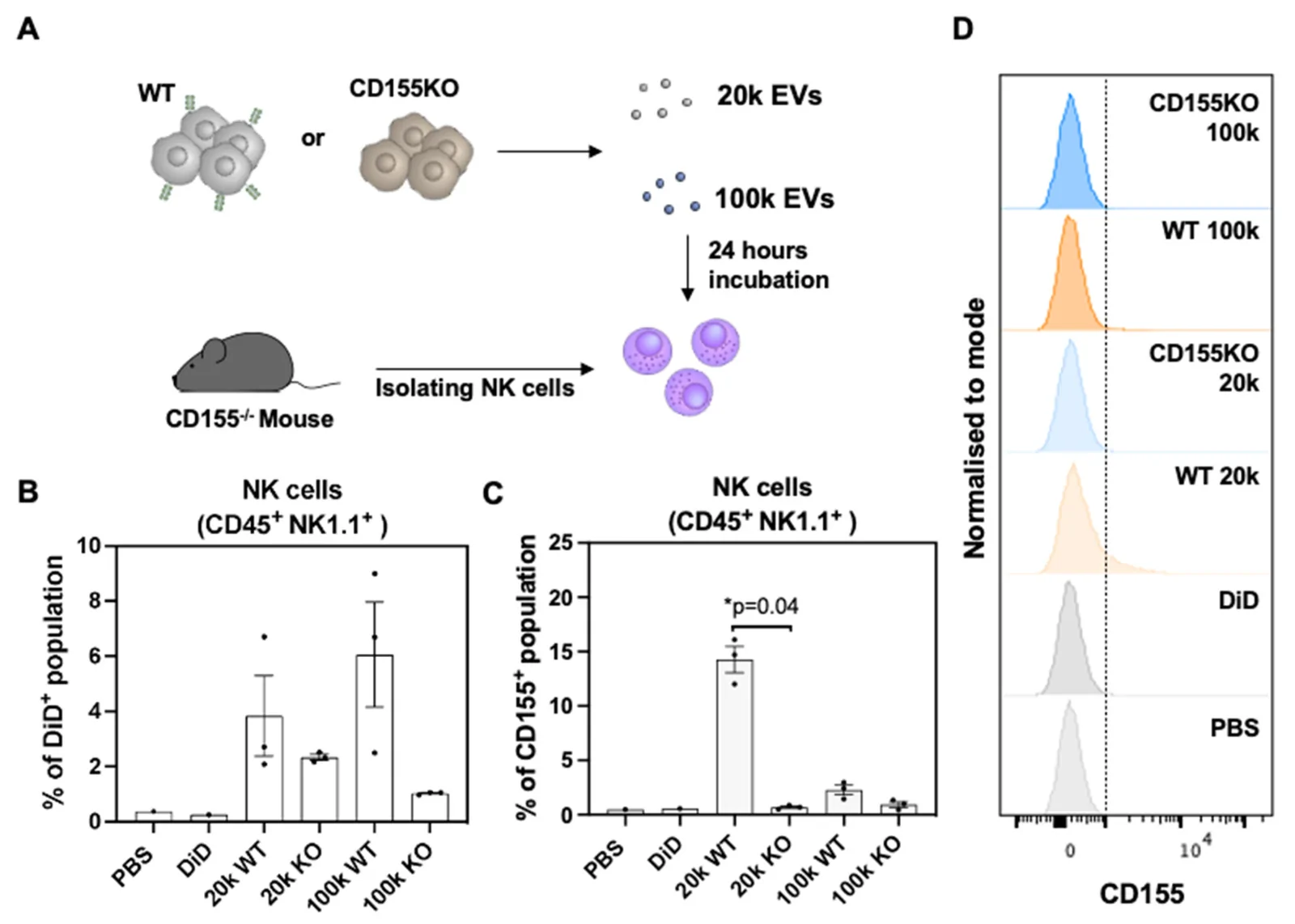
CD155 is transferred to primary murine NK cells via melanoma EVs. (**A**) Schematic of the EV uptake experiment in primary NK cells. Briefly, primary NK cells were isolated from the spleens of CD155^−/−^ mice by negative selection (see Section 4) and cultured overnight in IL-2-containing medium. Cells were then treated with 30 µg of DiD-labelled B16F10-WT (WT) or B16F10-CD155KO (CD155KO) 20k or 100k EVs for 24 h and analysed by flow cytometry (n = 3 biologically independent samples per group). PBS and DiD alone were used as controls. (**B**) Frequency of DiD^+^ cells within the CD45^+^NK1.1^+^ cell population. (**C**) Frequency of CD155^+^ cells within the CD45^+^NK1.1^+^ NK cell population. (**D**) Representative histograms showing CD155 expression within CD45^+^NK1.1^+^ NK cells. * *p* < 0.05 as analysed by the two-tailed Mann–Whitney U-test.

To test whether CD155 was transferred to immune cells via EVs, we next examined the uptake of B16F10-derived EVs by murine NK cells ex vivo. Primary NK cells were isolated from the spleens of CD155^−/−^ mice by negative selection (see Section 4; Figure S10). After isolation, NK cells were cultured overnight in IL-2-containing medium and then incubated for 24 h with DiD-labelled B16F10-WT or B16F10-CD155KO 20k or 100k EVs (Figure 3A). Both 20k and 100k B16F10-derived EVs were taken up by primary NK cells, independent of CD155 status, as shown by the increased frequency of DiD^+^ cells compared with DiD-alone controls (Figure 3B). However, acquisition of CD155 by CD155^−/−^ NK cells was detected only after incubation with B16F10-WT EVs, particularly B16F10-WT 20k EVs (Figure 3C,D). Notably, no significant differences were observed in the frequencies of DNAM1^+^ cells (Figure S11A) or TIGIT^+^ cells (Figure S11B). Together, these data show that CD155 is enclosed within B16F10-WT EVs and transferred to murine NK cells, with 20k EVs showing the stronger effect.

### 2.4. CD155-Enriched Melanoma-Derived 20k EVs Downregulate DNAM-1 Expression on Human NK Cells

**Figure 4:**
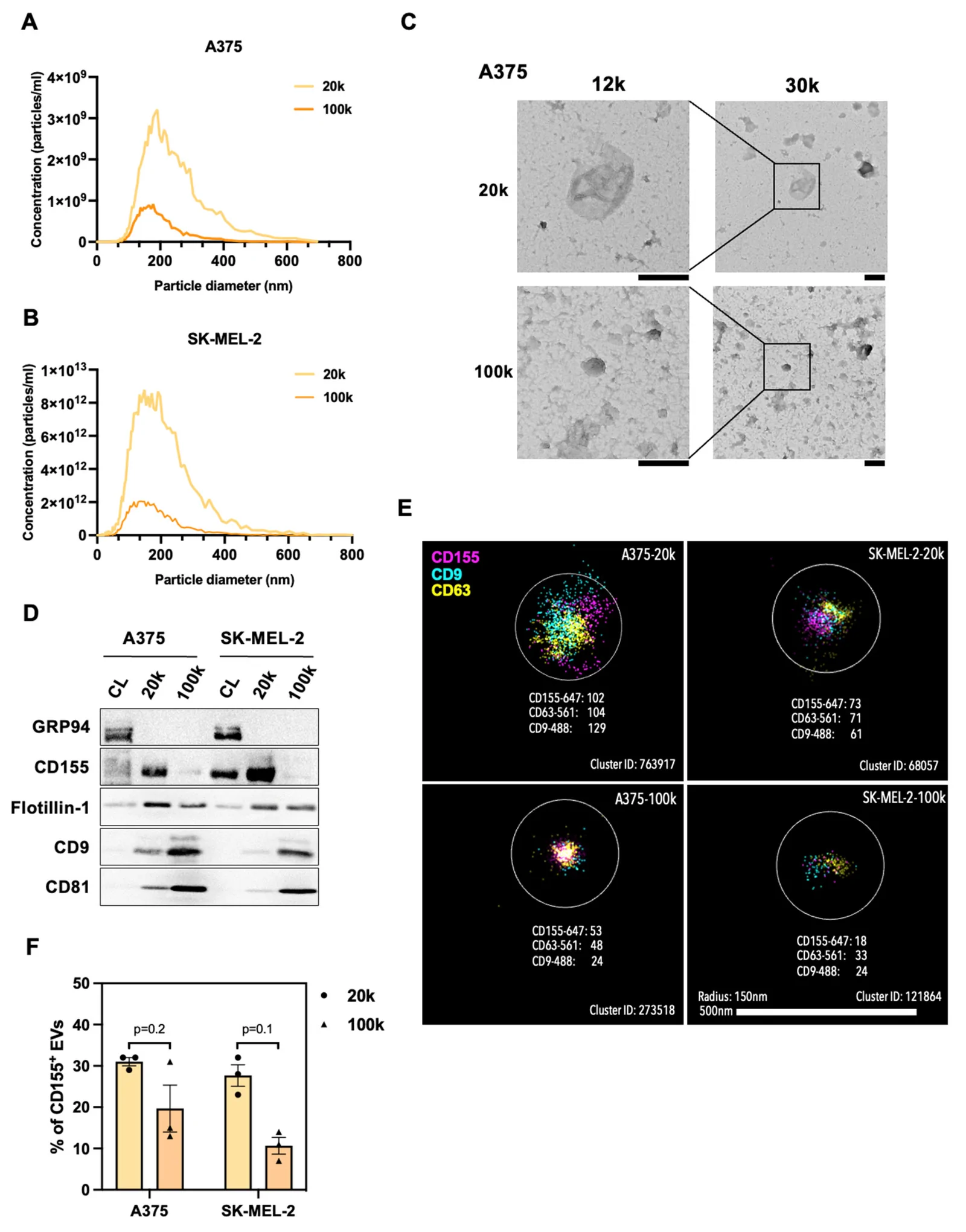
CD155 is enriched in human melanoma cell line-derived 20k EVs. The size distribution of A375-derived (**A**) and SK-MEL-2-derived (**B**) 20k and 100k EVs was analysed by nanoparticle tracking analysis. (**C**) The morphology was analysed by TEM. Representative images were acquired at 12,000× and 30,000× magnification using an accelerating voltage of 100 kV and a beam current of 10 μA (scale bar, 200 nm). (**D**) Representative immunoblots of EV markers (CD81, 25 kDa; CD9, 25 kDa; flotillin-1, 48 kDa) and the cell marker (GRP94, 94 kDa), as well as CD155 (human CD155, 70 kDa), in cell lysates (CL) and EVs isolated from conditioned media of A375 and SK-MEL-2 cells. Equal amounts of cell lysate (5 µg) and EV protein (2 × 10^9^ particles) were loaded to assess protein expression. Similar results were obtained from at least three biologically independent cell preparations. Source data are provided as a Source Data file. (**E**,**F**) 20k and 100k EVs derived from A375 or SK-MEL-2 cells were stained with antibodies against CD63 (magenta), CD155 (light blue), and CD9 (yellow), then captured on an S4 microfluidic slide from the EV Profile kit (ONI). Triple-positive EVs were identified in (**E**) and quantified in (**F**) using CODI software (ONi-bio, https://alto.codi.bio/ (accessed on 27 September 2026)) and manual verification. Results were obtained from at least two biologically independent cell preparations, and representative confocal images were acquired from at least six different fields. Scale bar, 500 nm.

Given the transfer of CD155 via B16F10-WT EVs, we next asked whether human melanoma-derived EVs show a similar pattern and thereby modulate human NK cells. CD155 has also been detected on EVs isolated from melanoma patient samples, where it correlated with CD8+ T cell dysfunction [46]. Because mouse and human CD155 isoforms differ, we used the human melanoma cell lines A375 and SK-MEL-2 to validate the function of melanoma CD155-containing EVs. The size distribution of 20k EVs was broader than that of 100k EVs derived from A375 and SK-MEL-2 cells (Figure 4A,B). Transmission electron microscopy showed EVs with diameters of approximately 200 nm in the 20k and 100k EV fractions (Figure 4C). A375- and SK-MEL-2-derived EVs were enriched in flotillin-1 in the 20k fraction, whereas CD9 and CD81 were more abundant in 100k EVs (Figure 4D). In addition, CD155 was highly enriched in EVs compared with cell lysates across all cell lines tested (B16F10, A375, and SK-MEL-2) (Figure 2C and Figure 4D). Direct stochastic optical reconstruction microscopy (dSTORM) also confirmed CD155 expression in A375- and SK-MEL-2-derived 20k and 100k EVs (Figure 4E). Approximately 30% of 20k EVs were CD155 positive, whereas only 10–20% of 100k EVs were CD155 positive (Figure 4F).

**Figure 5:**
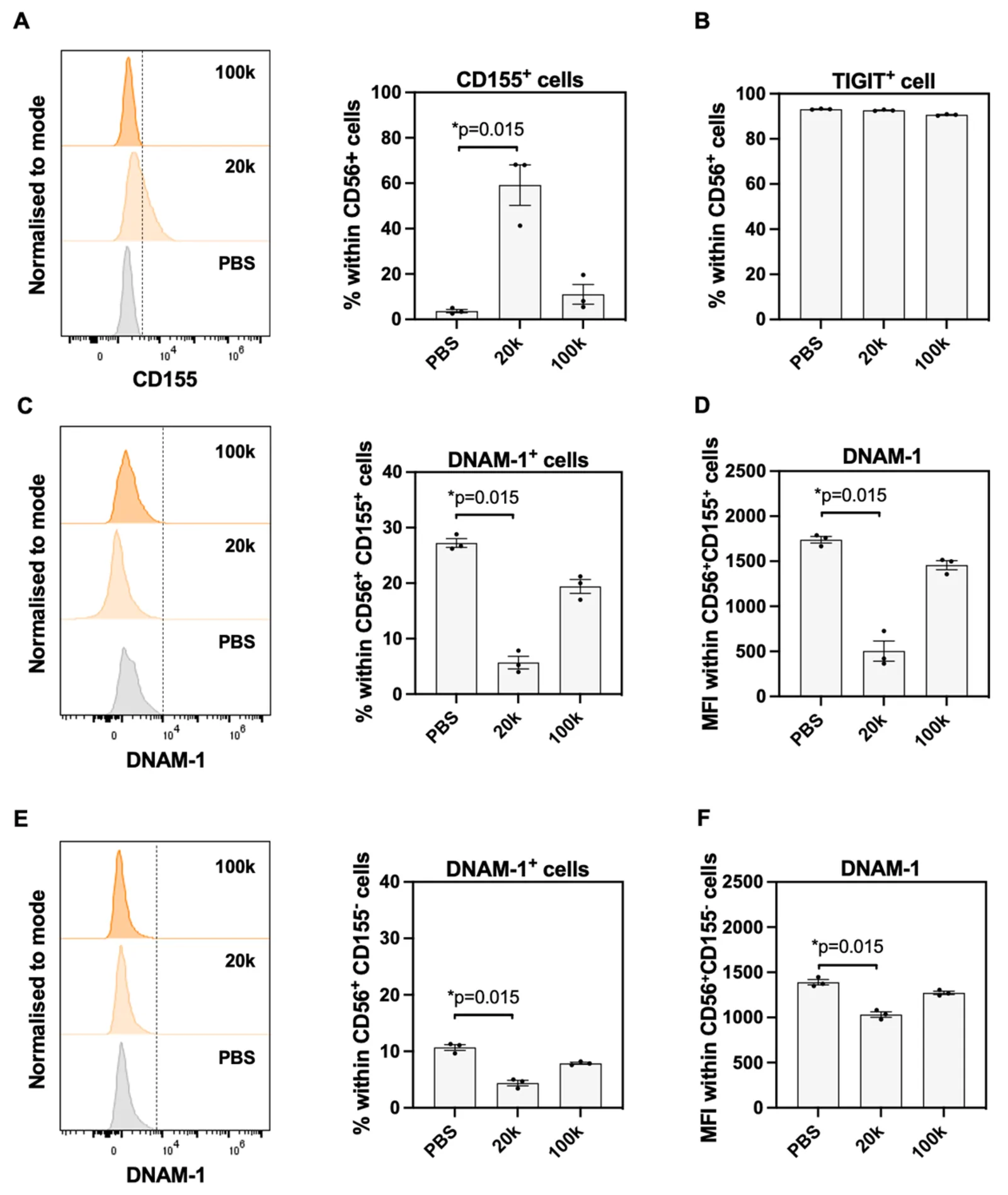
Increase in CD155 levels mediated by A375-derived EVs is associated with decreased DNAM-1 expression on NK-92MI cells. NK-92MI human NK cells were treated with either 10^10^ 20k EVs or 100k EVs for 24 h and then analysed by flow cytometry (n = 3 biologically independent samples per group). PBS was used as a negative control. (**A**) Histograms showing CD155 expression (**left**) and the frequency of CD155^+^ cells within the CD56^+^ NK92-MI population (**right**). (**B**) Frequency of TIGIT^+^ cells within CD56^+^ NK-92MI population. (**C**) Histograms showing DNAM-1 expression (**left**) and the frequency of DNAM-1^+^ cells within the CD56^+^CD155^+^ NK-92MI population (**right**). (**D**) Mean fluorescence intensity (MFI) of DNAM-1 in CD56^+^CD155^+^ NK-92MI cells. (**E**) Histograms showing expression of DNAM-1 (**left**) and frequency of DNAM-1^+^ cells within CD56^+^CD155^−^ NK-92MI cells (**right**). (**F**) Mean fluorescence intensity (MFI) of DNAM-1 in CD56^+^CD155^−^ NK-92MI cells. Data are presented as mean ± SEM. * *p* < 0.05, as analysed by the Kruskal–Wallis test.

We then assessed CD155 expression on NK-92MI human NK cells after 24 h incubation with A375-derived EVs, including both 20k and 100k fractions. Because NK-92MI cells require specialised culture conditions, we first confirmed that culture in EV-depleted medium (EDM) did not alter NK cell phenotype. The expression of CD56, DNAM-1 and TIGIT in EDM-cultured NK cells was similar to that in cells cultured under standard conditions (see Section 4; Figure S12). Remarkably, CD155 expression increased strongly on NK cells incubated with A375 20k EVs, whereas only a modest increase was observed after incubation with 100k EVs (Figure 5A). Consistent with the murine data, TIGIT expression was unchanged after incubation with CD155-containing EVs (Figure 5B). In contrast, DNAM-1 surface expression was significantly reduced on CD155^+^ NK cells after incubation with 20k EVs (Figure 5C,D). Notably, CD155 transfer from A375 melanoma 20k EVs to human NK-92MI cells was more robust (59.2 ± 8.9% of CD155^+^ cells) than transfer from B16F10-WT 20k EVs to primary murine NK cells (Figure 3C; 14.3 ± 2.1% of CD155^+^ cells), which may account for the different DNAM-1 outcomes observed in the murine uptake experiment (Figure S11 vs. Figure 5C,D). Finally, we also observed a slight but significant reduction in DNAM-1 surface expression in CD155^−^ NK cells after exposure to A375-derived EVs (Figure 5E,F). This may result from CD155 on 20k EVs engaging receptors on the NK cell surface prior to transfer of CD155 or from a putative crosstalk between CD155^−^ and CD155^+^ NK cells simultaneously present in the NK cell culture.

**Figure 6:**
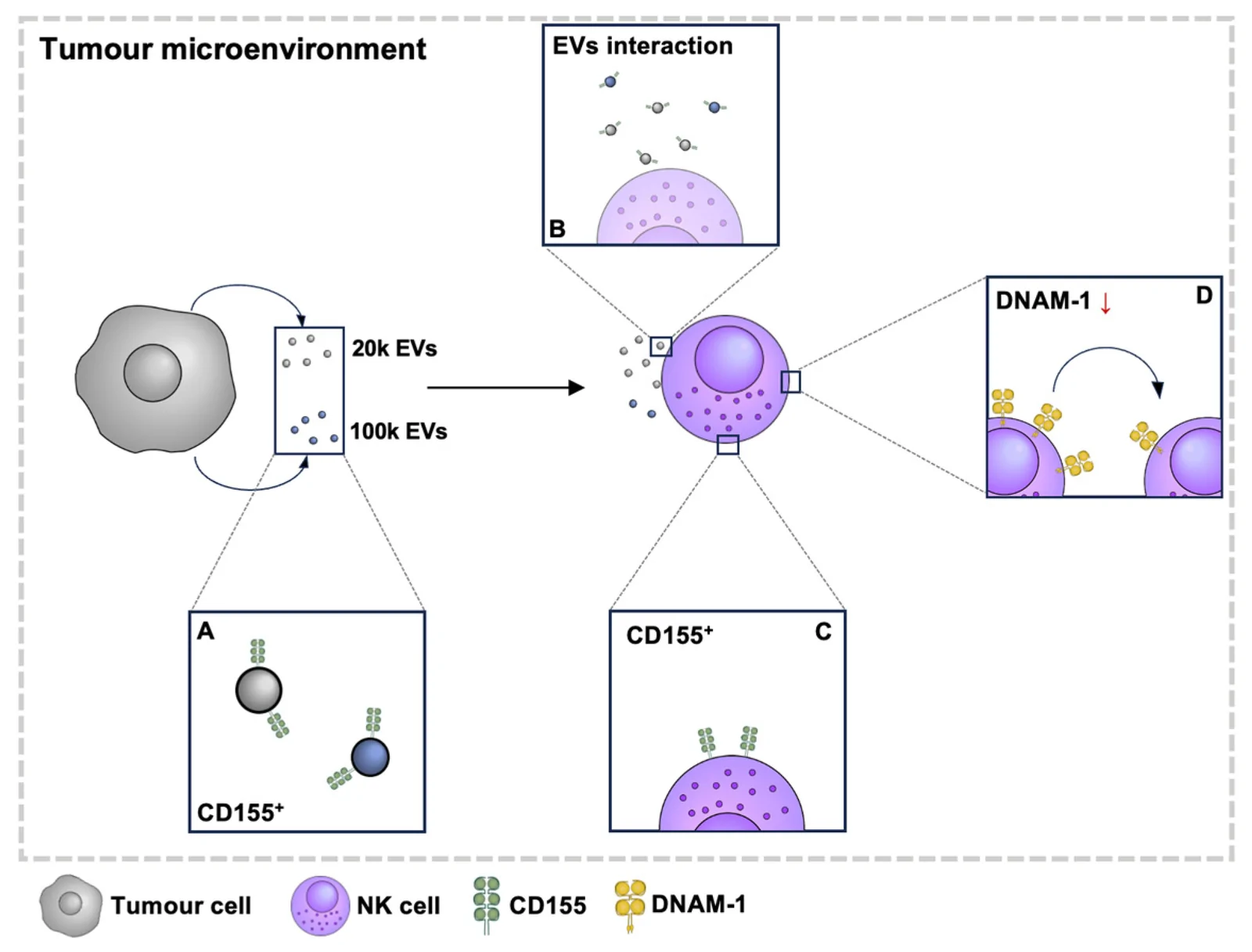
Summary of findings. (**A**) CD155 is enclosed within cancer-derived EVs, including 20k and 100k EVs, and is transferred to NK cells in the tumour microenvironment. (**B**) Uptake of EVs, particularly the 20k subset, by NK cells (**C**) induces CD155 expression on the surface of recipient cells and (**D**) suppresses DNAM-1 expression.

Overall, this study demonstrates that melanoma cells release CD155 via EVs, as shown in both mouse and human melanoma cell lines. Importantly, CD155 was significantly enriched in melanoma-derived EVs, particularly in the 20k fraction, compared with melanoma cell lysates. We further show that CD155 can be transferred from melanoma cells to immune cells through EVs and that this transfer is associated with reduced DNAM-1 expression on NK cells. Because DNAM-1 is a key activating receptor involved in NK cell-mediated anti-tumour immunity, these findings highlight a previously unrecognised mechanism by which melanoma-derived EVs may reprogram immune responses and contribute to tumour progression (Figure 6).

## 3. Discussion

Overexpression of CD155 on cancer cells has been shown to suppress activation of both CD8^+^ T and NK cells in the tumour microenvironment, thereby contributing to resistance to immunotherapy [11,24,26]. In this study, we identify a new mechanism by which CD155 is packaged into melanoma EVs and transferred to NK cells, where it downregulates the activating receptor DNAM-1. The interaction between cancer-derived EVs and immune cells can modulate immune responses and thereby influence melanoma progression [52,53,54,55]. More specifically, melanoma cell-derived EVs isolated from patient plasma have been shown to suppress NK cell activation through downregulation of the activating receptor NKG2D [56,57]. Our findings raise the possibility that melanoma EVs regulate NK cells through multiple activating receptors. Because both NKG2D and DNAM-1 are critical for NK cell-mediated lysis of melanoma cells [58,59], EV-mediated downregulation of DNAM-1 may cooperate with, or precede, reduced NKG2D expression to further impair NK cell function.

Both CD155 and CD112 can engage DNAM-1 and TIGIT, while CD112 also binds the inhibitory receptor CD112R/PVRIG [14,60,61]. The functional outcome of CD155–DNAM-1 engagement is context dependent [28]. During acute target-cell recognition, DNAM-1 ligation can cooperate with LFA-1 and other activating receptors to promote NK cell and CD8^+^ T cell degranulation, cytokine production and cytotoxicity [18,19]. Notably, our study suggests that cancer-derived CD155^+^ EVs downregulate DNAM-1 expression on NK cells in the tumour microenvironment. Previous studies have shown that CD155 on tumour cells induces DNAM-1 internalisation and degradation in CD8^+^ T and NK cells [11,24,62]. High CD155 surface expression on tumour cells can trigger DNAM-1 degradation through Src kinase-mediated phosphorylation, CBL-B-dependent ubiquitination, and subsequent proteasomal degradation [24]. Thus, although acute CD155–DNAM-1 engagement can be activating, persistent exposure to CD155-high tumour cells or tumour-derived CD155^+^ EVs may ultimately diminish DNAM-1-dependent activation [24,28]. Our findings show that CD155 is also markedly enriched in melanoma-derived EVs and may therefore impair DNAM-1 signalling through a similar mechanism. Furthermore, TIGIT is an important inhibitory immune receptor that regulates T cell and NK cell function [63,64], and TIGIT deletion in human NK cells enhances killing in vitro [65,66]. Because CD155 binds TIGIT with higher affinity than DNAM-1 and has been shown to inhibit NK cell function, transfer of melanoma-derived CD155 via EVs may also exacerbate NK cell dysfunction through TIGIT engagement [15,62,67]. Notably, although CD155 engagement can also induce TIGIT internalization, TIGIT appears to be preferentially recycled back to the cell surface rather than degraded, unlike DNAM-1, potentially allowing sustained TIGIT-mediated inhibitory signalling despite repeated CD155 exposure [11]. Additionally, TIGIT may also interact directly with DNAM-1 in cis on the same immune cell, disrupting DNAM-1 homodimerization and attenuating its co-stimulatory function [62]. These mechanisms may collectively shift the balance from DNAM-1-mediated activation towards TIGIT-dominated inhibition in CD155-rich tumour microenvironments.

The CD155 protein is expressed as multiple alternatively spliced isoforms, including membrane-anchored and soluble forms [48]. The biological effects of CD155 may depend on its physical form. Membrane-bound CD155 on tumour cells can support multivalent engagement of DNAM-1 during direct cell–cell contacts and may promote immune cell activation under acute conditions. However, sustained exposure to CD155-high tumour cells can induce DNAM-1 downregulation [24]. In contrast, soluble CD155 has been reported to inhibit DNAM-1-dependent NK cell activity, potentially by competitively binding DNAM-1 and limiting its interaction with membrane-bound ligands [68]. Soluble CD155 has been detected in human, but not in murine models [68,69]. Because EVs are membrane-enclosed particles, the CD155 detected in our EV fractions is expected to be predominantly membrane-associated and is therefore consistent with incorporation of transmembrane CD155 isoforms. Although our mouse experiments focused on tumour cell-associated and EV-enriched CD155 fractions, a contribution of soluble CD155 cannot be fully excluded in the experiments using human melanoma cell lines. The 20k EV fraction was isolated by centrifugation at 20,000× *g*, a condition expected to retain most freely soluble proteins in the supernatant while enriching larger extracellular particles in the pellet [31]. The 100k fraction was isolated by centrifugation at 100,000× *g* and subsequently purified by size exclusion chromatography to reduce contamination by freely soluble proteins [31].

In this study, CD155 was enriched in the 20k EV fractions derived from both mouse and human melanoma cell lines, which may contribute to the more pronounced reduction in DNAM-1 expression observed after treatment with 20k EVs. In human melanoma cell lines, dSTORM analysis indicated that the 20k fraction contained a greater proportion of CD155-positive EVs and exhibited higher CD155 abundance than the 100k fraction. The 20k fraction also displayed a broader particle-size distribution, with larger particles and was relatively enriched in flotillin-1, whereas CD9 and CD81 were more abundant in the 100k fraction. Therefore, the stronger CD155 transfer and DNAM-1 downregulation induced by 20k EVs may reflect higher CD155 cargo and differences in EV uptake and intracellular trafficking.

A limitation of this study is that the in vivo model cannot distinguish EV-mediated CD155 transfer from contact-dependent membrane transfer, including trogocytosis [70,71]. Although our data demonstrate that CD155 was transferred to NK cells after treatment with CD155-EVs in vitro, the relative contribution of EVs and trogocytosis to CD155 transfer within the tumour microenvironment remains to be determined. Future studies using direct co-culture versus trans-well separation, tumour-membrane labelling, and/or inhibition of EV release or uptake will be required to define the relative contributions of these mechanisms.

Current research has mainly focused on CD155 receptors on NK cells and their interaction with CD155 on tumour cells [11,72]. However, little is known about the role of CD155 expression and potential signalling within NK cells themselves. CD155 contains an ITIM-like domain, which has been implicated in several processes in tumour cells [73,74]. How surface CD155 influences NK cell phenotype and function remains unclear. Therefore, our finding that melanoma cells transfer CD155 to NK cells warrants further investigation into the capacity and consequences of CD155 signalling in NK cells.

In the tumour microenvironment, NK cells play a central role in anti-tumour immune responses. The interaction between CD155 and DNAM-1 on NK cells promotes activation, cytokine secretion and anti-tumour immunity [20,75]. However, CD155 overexpression impairs NK cell activation [68,73,76] and cytokine secretion, an effect that can be reversed by TIGIT inhibition [11,27]. Restoring NK cell function remains a major goal of NK cell-based immunotherapy [11,77,78]. EVs isolated from irradiated B16F10 melanoma cells have previously been shown to enhance NK cell activation and cytokine secretion, thereby delaying melanoma tumour growth [79]. In addition, CAR-T cell therapy targeting CD155 has recently been shown to reduce tumour burden [80]. Overall, our findings may provide a mechanistic basis for the development of CD155-targeted therapies.

## 4. Materials and Methods

### 4.1. Mice

CD155^−/−^ C57Bl/6 mice were kindly provided by Professor Yoshimi Takai (Kobe University, Japan) [81]. Male mice were used at 6–10 weeks of age and bred and maintained at the QIMR Berghofer Medical Research Institute. Mice were housed at the QIMR Berghofer Biological Resources Facility under a controlled light cycle, at a density of one to six animals per cage; all cage-mates were born on the same date, and mice housed singly were provided with additional environmental enrichment (a cardboard roll). Animal health was monitored regularly by on-site veterinary staff. Mice were not allocated to groups using formal randomisation software; instead, mice born on the same date (typically cage-mates) were alternately distributed between the WT and CD155KO groups to balance age across groups. Blinding of investigators to group allocation was not performed at any stage of this study, including tumour cell injection, tumour measurement, flow cytometric acquisition and analysis, and data analysis. Mice had not undergone any previous procedures prior to enrolment in this study. All animal procedures were conducted in accordance with Australian National Health and Medical Research regulations on the use and care of experimental animals and approved by the QIMR Berghofer Medical Research Institute Animal Ethics Committee (A2109-626, P3759, approved 14 October 2021).

### 4.2. Cell Lines and Cell Culture

Mouse melanoma B16F10 cells were maintained as previously described [82]. Mouse melanoma CD155 knock-out cells (B16F10-CD155KO) were previously generated by CRISPR-Cas9 in collaboration with Professor Bald’s laboratory, previously at the QIMR Berghofer Medical Research Institute [24]. The human melanoma cell lines A375 (ATCC CRL-1619) and SK-MEL-2 (ATCC HTB-68), as well as the human NK cell line NK-92MI (ATCC CRL-2408), were purchased from the American Type Culture Collection (ATCC, Manassas, VA, USA). Cells were cultured in Dulbecco’s Modified Eagle Medium (DMEM; Gibco^TM^, Waltham, MA, USA, cat. no. 11995073) supplemented with 5% fetal bovine serum (FBS; Gibco^TM^, cat. no. 10099141) and 1% penicillin–streptomycin (P/S; Gibco^TM^, cat. no. 15140122) in humidified incubators at 37 °C with 5% CO_2_. B16F10 cells were additionally supplemented with 1% GlutaMAX (Gibco^TM^, cat. no. 35050061). CD155 expression on melanoma cells was routinely analysed by flow cytometry. Briefly, cells were harvested and washed with PBS containing 2% FBS before being stained with the relevant antibodies for 20 min. Flow cytometric acquisition was performed using an LSRFortessa™ (BD Biosciences, Franklin Lakes, NJ, USA) and the BD FACSDiva™ software (BD Biosciences, v8), and analysis was performed using the FlowJo software (Tree Star, v10.7.1).

Natural killer NK-92MI cells were obtained in collaboration with Professor Choi’s laboratory at CHA University in South Korea [83]. NK-92MI cells were cultured in Minimum Essential Medium α (α-MEM, Gibco^TM^, cat. no. 12571063) supplemented with 12.5% horse serum (Sigma-Aldrich, St. Louis, MO, USA, cat. no. H1138), 12.5% FBS, 0.2 mM myo-Inositol (Sigma-Aldrich, cat. no. I7508), 0.1 mM 2-Mercaptoethanol (Gibco^TM^, cat. no. 31350010) and 0.02 mM folic acid (Sigma-Aldrich, cat. no. F8758).

### 4.3. Antibodies

The following primary and secondary antibodies were used for Western blotting: rabbit anti-CD9 (1:1000 dilution; abcam, cat. no. ab223052), rabbit anti-GM130 (1:1000 dilution; abcam, cat. no. ab52649), mouse anti-HSP70 (1:1000 dilution; BD Biosciences, cat. no. BD610608), mouse anti-Flotilin-1 (1:1000 dilution; BD Biosciences, cat. no. BD610821), rat anti-CD155 (1:2000 dilution; Biolegend, cat. no. 131502), mouse anti-CD81 (1:1000 dilution; Santa Cruz, Dallas, TX, USA, cat. no. sc166029), rabbit anti-GRP94 (1:1000 dilution; Abcam, Cambridge, UK, cat. no. ab238126), goat anti-mouse HRP-conjugated (1:30,000 dilution; Pierce, Rockford, IL, USA, cat. no. 1858413), goat anti-rabbit HRP-conjugated (1:10,000 dilution; Pierce, cat. no. 1858415), and goat anti-rat HRP-conjugated (1:3000 dilution; R&D Systems, Minneapolis, MN, USA, cat. no. HAF005).

The following fluorochrome-conjugated antibodies were used for flow cytometry: anti-MHCII BUV395 (1:400 dilution; BD Biosciences, cat. no. 743876), anti-CD4 BV510 (1:300 dilution; BD Biosciences, cat. no. 563106), anti-CD11c BV650 (1:100 dilution; BD Biosciences, cat. no. 564079), anti-CD62L BV650 (1:200 dilution; BD Biosciences, cat. no. 504168), anti-CD45.2 BV786 (1:100 dilution; BD Biosciences, cat. no. 563686), anti-Ly6C FITC (1:300 dilution; BD Biosciences, cat. no. 553104), anti-Ly6G PE (1:400 dilution; BD Biosciences, cat. no. 220461), anti-CD155 APC (1:200 dilution; eBioscience, San Diego, CA, USA, cat. no. 17-1551-82), anti-CD3 APC (1:200 dilution; eBioscience, cat. no. 17-0031-82), anti-CD19 APC-e780 (1:100 dilution; eBioscience, cat. no. 47-0193-80), anti-F4/80 APC-e780 (1:200 dilution; eBioscience, cat. no. 47-4801-80), anti-CD69 e450 (1:100 dilution; eBioscience, cat. no. 48.0691.80), anti-Ly6G e450 (1:100 dilution; eBioscience, cat. no. 48.5931.80), anti-CD11b FITC (1:300 dilution; eBioscience, cat. no. 11-0112-81), anti-CD3 FITC (1:200 dilution; eBioscience, cat. no. 11-0031-82), anti-CD44 FITC (1:100 dilution; eBioscience, cat. no. 11-0441-81), anti-CD11b PE-Cy7 (1:1000 dilution; eBioscience, cat. no. 25-0112-81), anti-CD8a PE-Cy7 (1:300 dilution; eBioscience, cat. no. 25-0081-81), anti-NK1.1 PE-Cy7 (1:100 dilution; eBioscience, cat. no. 25-5941-82), anti-NKG2D PerCP-e710 (1:100 dilution; eBioscience, cat. no. 46-5882-82), anti-CD155 PE (1:300 dilution; Biolegend, San Diego, CA, USA, cat. no. 131508), anti-CD155 AF647 (1:1000 dilution; Biolegend, cat. no. 131528), anti-Ly49D AF647 (1:200 dilution; Biolegend, cat. no. 138306), anti-TIGIT BV421 (1:100 dilution; Biolegend, cat. no. 142111), anti-Ly49A Pacific Blue (1:200 dilution; Biolegend, cat. no. 116810), anti-CD25 PE (1:300 dilution; Biolegend, cat. no. 102008), anti-DNAM-1 PE (1:200 dilution; Biolegend, cat. no. 132006), anti-NK1.1 PerCP-Cy5.5 (1:200 dilution; Biolegend, cat. no. 108728).

The following fluorochrome-conjugated antibodies were used for immunofluorescence: anti-CD155 PE (1:100 dilution; Biolegend, cat. no. 131508), anti-CD11b FITC (1:100 dilution; eBioscience, cat. no. 11-0112-81).

### 4.4. Western Blot Analysis

Cultured cells were rinsed with DPBS and harvested using 0.25% EDTA-trypsin. Cells were pelleted and lysed in RIPA buffer containing 1% NP-40, 0.5% sodium deoxycholate, 0.1% SDS, 150 mM sodium chloride and 50 mM Tris HCL, pH 8.0. Bradford reagent (Bio-Rad, Hercules, CA, USA, cat. no. 5000205) was used to determine protein concentration in both cell lysates and EV suspensions according to the manufacturer’s instructions. Samples were then mixed with loading buffer containing 10% SDS, 1 M Tris-HCl, 30% glycerol, 5% 2-mercaptoethanol and 0.12% bromophenol blue, boiled at 95 °C for 5 min, separated by 10% SDS-PAGE, and transferred onto PVDF membranes. Membranes were blocked in 5% non-fat dry milk diluted in PBS-T for 1 h and then incubated with the relevant antibodies. Signal was detected by using enhanced chemiluminescence (ECL; Cytiva, Marlborough, MA, USA, cat. no. GEHERPN2232).

### 4.5. In Vivo Tumour Model

B16F10 or B16F10-CD155KO cells (5 × 10^5^) were subcutaneously injected into the flanks of CD155^−/−^ C57Bl/6 mice. Body weight and tumour volume were measured every 2 days from day 5 post-tumour implantation onward. In accordance with the Australian Code for the Care and Use of Animals for Scientific Purposes, pre-defined humane endpoints for early euthanasia were a tumour volume exceeding 500 mm^3^ or body weight loss exceeding 20%, whichever occurred first. No mice reached these humane endpoints before the scheduled study endpoint, and no unexpected adverse events were observed. On day 14 post-injection, mice were culled, and tumours and spleens were harvested for further analysis. No animals or data points were excluded from this study, analysis, or presented results. Sample sizes were determined primarily by the availability of mice supplied by the breeding service; each experiment included a minimum of three mice per group, with the exact number of mice used in each experiment specified in the corresponding figure legend.

### 4.6. Tissue Processing for Flow Cytometry Analysis

Tumours, spleens, and lymph nodes were collected, transferred to 6-well plates containing DMEM supplemented with 5% FBS and 1% P/S, and kept on ice for subsequent steps. Tumour tissues were minced and digested with 2 mg/ml collagenase type IV (Worthington Biochemical Corp, Lakewood, NJ, USA, cat. no. LS004189) under agitation at 37 °C for 10 min. Tumour tissue samples and spleens were passed through a 70-µm cell strainer (BD Biosciences) to obtain single-cell suspensions. To remove red blood cells, cell preparations were treated with ammonium chloride lysis buffer. Cell pellets were resuspended in 3 mL of FACS buffer and re-filtered through Falcon^®^ tubes with 35-µm cell strainer caps (cat. no. 352235). Samples were then centrifuged at 500× *g* for 5 min, and the cell pellets were resuspended in FACS buffer for staining. Cells were incubated with the relevant antibodies for 20 min on ice and fixed in BD Stabilizing Fixative buffer (cat. no. 338036). Zombie Yellow or Zombie Aqua (1:300 dilution; BioLegend, cat. nos. 423104 and no. 423101, respectively) was used as a viability dye. Flow cytometric acquisition was performed using an LSR-Fortessa^TM^ (BD Biosciences) and BD FACSDiva™ software (BD Biosciences, v8), and analysis was performed using FlowJo software (Tree Star, v10.7.1).

### 4.7. EV-Depleted Media (EDM) Preparation

Briefly, DMEM containing 40% FBS and 1% P/S was prepared and ultracentrifuged at 100,000× *g* at 4 °C for 16–20 h. The supernatant was collected, filtered through 0.22 μm Stericup filter devices (Merck Millipore, Darmstadt, Germany, cat. no. S2GPU02RE) and stored at 4 °C as EDM stock. For working medium, the 40% FBS EDM was diluted to 5% FBS EDM.

### 4.8. EV Isolation

Cells were seeded into 150 × 25 mm dishes at a density of 1 × 10^6^ cells/dish for the B16F10 WT line, 1.5 × 10^6^ cells/dish for B16F10-CD155KO cell line, and 1.5 × 10^6^ cells/dish for the A375 cell line. After overnight adhesion, cells were washed with Dulbecco’s phosphate-buffered saline (DPBS; Gibco™, cat. no. 14190-144) and incubated in 5% FBS EDM (14 mL/dish). Conditioned media were collected after 48 h. EVs were isolated as previously described [84] with slight modifications. Briefly, the medium was first centrifuged at 500× *g* for 10 min to remove cells and debris. Supernatants were transferred to ultracentrifuge tubes and centrifuged at 20,000× *g* for 1 h in a Beckman Coulter Optima™ XPN-100 Ultracentrifuge (Brea, CA, USA) with a 50.2Ti rotor. The pellet was collected as 20k EVs. Supernatants were then transferred to clean tubes and centrifuged at 100,000× *g* for 1 h 40 min to isolate 100k EVs. All pellets were resuspended in DPBS. 100k EVs were further purified by overlaying the EV suspension onto qEVoriginal 70-nm size exclusion columns (SEC; Izon Science Ltd., Christchurch, New Zealand). The fractions collected from qEV were further concentrated to a final volume of approximately 200 µL using Amicon Ultra-4 10-kDa nominal molecular weight centrifugal filter units (Merck Millpore).

### 4.9. Nanoparticle Tracking Analysis

Particle abundance and size distribution were assessed using the ZetaView system (Particle Metrix, Inning am Ammersee, Germany) and the corresponding software (version 8.05.16 SP3), as previously described [85] with slight modifications. Briefly, before sample measurement, the system temperature was set to 23 °C. Samples were diluted in PBS, which was filtered through 0.22 µm Stericup filter devices (Merck Millipore, cat. no. S2GPU02RE) to 1 mL before loading into the cell.

### 4.10. Transmission Electron Microscopy

Purified EVs were fixed with paraformaldehyde and applied to 200-mesh carbon-coated copper grids (01801, Ted Pella, Inc., Redding, CA, USA) for 5 min. The grids were then negatively stained with 10 μL of 4% uranyl acetate (22400, Electron Microscopy Sciences, Hatfield, PA, USA) for 2 min and washed with filtered water. After drying for 2 h, the grids were examined using a Hitachi HT7700 transmission electron microscope (Tokyo, Japan) operating at an accelerating voltage of 100 kV and an emission current of 10.2 μA.

### 4.11. EVs Confocal (ONi) Imaging

EVs were stained using the EV Profiler Kit (ONi, cat. no. EV-MAN-1.0) according to the manufacturer’s instructions. Direct stochastic optical reconstruction microscopy (dSTORM) images were acquired using an ONI Nanoimager (ONI-bio, Oxford, UK) and analysed using CODI software (ONi-bio, https://alto.codi.bio/ (accessed on 27 September 2026)).

### 4.12. DiD Labelling of EVs

EVs were fluorescently labelled using Vybrant^®^ DiD dye (Thermo Fisher Scientific/Life Technologies, Waltham, MA, USA, cat. no. V22887) according to the manufacturer’s instructions, with modifications [86].

### 4.13. EVs Incubation with NK Cells

Primary NK1.1^+^/CD3^−^ cells were isolated from whole spleens of CD155^−/−^ C57Bl/6 mice using the MagniSort™ Mouse NK Cell Enrichment Kit (Invitrogen™, Waltham, MA, USA, cat. no. 8804-6828-74) according to the manufacturer’s instructions. Isolated cells were cultured overnight in Roswell Park Memorial Institute (RPMI) 1640 medium (Gibco™, cat. no. 11875093) supplemented with 10% FBS (diluted from RPMI EDM stock), 0.05 mM 2-Mercaptoethanol, 1 mM sodium pyruvate (Gibco™, cat. no. 11360070), 10 mM HEPES (Gibco™, cat. no. 15630106), 1% P/S, and 1000 units/mL of IL-2 in humidified incubators at 37 °C with 5% CO_2_. NK cells were then treated with either 20k EVs or 100k EVs for 24 h. Cells were harvested and washed with PBS containing 2% FBS before being stained with the relevant antibodies for 20 min. Flow cytometric acquisition was performed using an LSRFortessa™ (BD Biosciences) and BD FACSDiva™ software (BD Biosciences, v8), and analysis was performed using FlowJo software (Tree Star, v10.7.1).

NK-92MI NK cells were seeded into 12-well plates at 1 × 10^5^ cells/well and cultured for 16 h. For EV incubation, the medium was changed to 12.5% FBS EDM supplemented with 0.2 mM myo-inositol, 0.1 mM 2-mercaptoethanol, 0.02 mM folic acid, and α-MEM. NK-92MI cells were then treated with either 20k EVs or 100k EVs for 24 h. Cells were harvested and washed with PBS containing 2% FBS before being stained with the relevant antibodies for 20 min. Flow cytometric acquisition was completed using a Cytoflex (Beckman Coulter, Brea, CA, USA) and CytExpert software (version 2.4), and analysis was performed using FlowJo software (TreeStar, v10.7.1).

### 4.14. Statistical Analysis

Data are presented as mean ± standard error of the mean (SEM) from at least three independent experiments. GraphPad Prism (v10.0; GraphPad Software) was used for data analysis; tumour growth curves were analysed by the Wilcoxon test, and mean differences were compared using the two-tailed Mann–Whitney U test or Kruskal–Wallis test. Statistical significance was set at *p* < 0.05. RStudio (v2023.12.0+369; The R Foundation for Statistical Computing) was used to run Spearman correlation tests and generate correlation plots. Non-parametric statistical tests were chosen a priori, as flow cytometry and tumour growth data are typically not normally distributed; formal normality testing was not performed.

## 5. Conclusions

In this study, we demonstrated that melanoma cells can transfer the immunomodulatory ligand CD155 to various immune cell populations within the tumour microenvironment. Mechanistically, we showed that EVs isolated from mouse and human melanoma cells contained CD155. In vitro incubation of CD155^+^ melanoma-derived EVs with NK cells increased CD155 expression on NK cells and concomitantly downregulated DNAM-1, corroborating our in vivo findings in mice. Altogether, these data indicate that CD155 present on tumour-derived EVs may modulate NK cell activity in cancer and thereby play an important role in tumour progression.

## Data Availability

All data associated with this study are present in the paper or in the Supporting Information.

## Supplementary Materials

The following supporting information can be downloaded at https://www.mdpi.com/article/10.3390/ijms27198699/s1.

## Abbreviations

The following abbreviations are used in this manuscript:

PVR	Poliovirus receptor
DNAM-1	DNAX accessory molecule-1
TIGIT	T cell immunoreceptor with Ig and ITIM domains
NK Cells	Natural killer cells
EVs	Extracellular vesicles
lEVs	Large extracellular vesicles
sEVs	Small extracellular vesicles
B16F10-WT	B16F10 wild type cells
B16F10-CD155KO	B16F10 CD155 knock-out cells
20k EVs	Extracellular vesicles pellet that was collected after spinning down cell conditioned medium at 20,000× *g*
100k EVs	Extracellular vesicles pellet that was collected after spinning down cell conditioned medium at 100,000× *g*
EDM	EV-Depleted Media
DCs	Dendritic cells
MDSCs	Myeloid-derived suppressor cells
dSTORM	Direct stochastic optical reconstruction microscopy

## References

[1] Fidler M.M., Gupta S., Soerjomataram I., Ferlay J., Steliarova-Foucher E., Bray F. (2017). Cancer incidence and mortality among young adults aged 20–39 years worldwide in 2012: A population-based study. Lancet Oncol..

[2] Alexandrov L.B., Nik-Zainal S., Wedge D.C., Aparicio S.A.J.R., Behjati S., Biankin A.V., Bignell G.R., Bolli N., Borg A., Børresen-Dale A.-L. (2013). Signatures of mutational processes in human cancer. Nature.

[3] Ralli M., Botticelli A., Visconti I.C., Angeletti D., Fiore M., Marchetti P., Lambiase A., de Vincentiis M., Greco A. (2020). Immunotherapy in the Treatment of Metastatic Melanoma: Current Knowledge and Future Directions. J. Immunol. Res..

[4] Schumacher T.N., Schreiber R.D. (2015). Neoantigens in cancer immunotherapy. Science.

[5] Wu L.Y., Park S.H., Jakobsson H., Shackleton M., Moller A. (2024). Immune Regulation and Immune Therapy in Melanoma: Review with Emphasis on CD155 Signalling. Cancers.

[6] Wolchok J.D., Chiarion-Sileni V., Gonzalez R., Grob J.J., Rutkowski P., Lao C.D., Cowey C.L., Schadendorf D., Wagstaff J., Dummer R. (2022). Long-Term Outcomes with Nivolumab Plus Ipilimumab or Nivolumab Alone Versus Ipilimumab in Patients with Advanced Melanoma. J. Clin. Oncol..

[7] Pires da Silva I., Ahmed T., Reijers I.L.M., Weppler A.M., Betof Warner A., Patrinely J.R., Serra-Bellver P., Allayous C., Mangana J., Nguyen K. (2021). Ipilimumab alone or ipilimumab plus anti-PD-1 therapy in patients with metastatic melanoma resistant to anti-PD-(L)1 monotherapy: A multicentre, retrospective, cohort study. Lancet Oncol..

[8] Wolchok J.D., Chiarion-Sileni V., Rutkowski P., Cowey C.L., Schadendorf D., Wagstaff J., Queirolo P., Dummer R., Butler M.O., Hill A.G. (2025). Final, 10-Year Outcomes with Nivolumab plus Ipilimumab in Advanced Melanoma. N. Engl. J. Med..

[9] van Not O.J., Blokx W.A.M., van den Eertwegh A.J.M., de Meza M.M., Haanen J.B., Blank C.U., Aarts M.J.B., van den Berkmortel F., de Groot J.W.B., Hospers G.A.P. (2022). BRAF and NRAS Mutation Status and Response to Checkpoint Inhibition in Advanced Melanoma. JCO Precis. Oncol..

[10] Gide T.N., Quek C., Menzies A.M., Tasker A.T., Shang P., Holst J., Madore J., Lim S.Y., Velickovic R., Wongchenko M. (2019). Distinct Immune Cell Populations Define Response to Anti-PD-1 Monotherapy and Anti-PD-1/Anti-CTLA-4 Combined Therapy. Cancer Cell.

[11] Chauvin J.M., Ka M., Pagliano O., Menna C., Ding Q., DeBlasio R., Sanders C., Hou J., Li X.Y., Ferrone S. (2020). IL15 Stimulation with TIGIT Blockade Reverses CD155-mediated NK-Cell Dysfunction in Melanoma. Clin. Cancer Res..

[12] Ziogas D.C., Theocharopoulos C., Koutouratsas T., Haanen J., Gogas H. (2023). Mechanisms of resistance to immune checkpoint inhibitors in melanoma: What we have to overcome?. Cancer Treat. Rev..

[13] Kucan Brlic P., Lenac Rovis T., Cinamon G., Tsukerman P., Mandelboim O., Jonjic S. (2019). Targeting PVR (CD155) and its receptors in anti-tumor therapy. Cell Mol. Immunol..

[14] Sanchez-Correa B., Valhondo I., Hassouneh F., Lopez-Sejas N., Pera A., Bergua J.M., Arcos M.J., Banas H., Casas-Aviles I., Duran E. (2019). DNAM-1 and the TIGIT/PVRIG/TACTILE Axis: Novel Immune Checkpoints for Natural Killer Cell-Based Cancer Immunotherapy. Cancers.

[15] Stanietsky N., Simic H., Arapovic J., Toporik A., Levy O., Novik A., Levine Z., Beiman M., Dassa L., Achdout H. (2009). The interaction of TIGIT with PVR and PVRL2 inhibits human NK cell cytotoxicity. Proc. Natl. Acad. Sci. USA.

[16] Bevelacqua V., Bevelacqua Y., Candido S., Skarmoutsou E., Amoroso A., Guarneri C., Strazzanti A., Gangemi P., Mazzarino M.C., D’Amico F. (2012). Nectin like-5 overexpression correlates with the malignant phenotype in cutaneous melanoma. Oncotarget.

[17] Zhang Z., Wu N., Lu Y., Davidson D., Colonna M., Veillette A. (2015). DNAM-1 controls NK cell activation via an ITT-like motif. J. Exp. Med..

[18] Tahara-Hanaoka S., Shibuya K., Onoda Y., Zhang H., Yamazaki S., Miyamoto A., Honda S., Lanier L.L., Shibuya A. (2004). Functional characterization of DNAM-1 (CD226) interaction with its ligands PVR (CD155) and nectin-2 (PRR-2/CD112). Int. Immunol..

[19] Shibuya K., Shirakawa J., Kameyama T., Honda S., Tahara-Hanaoka S., Miyamoto A., Onodera M., Sumida T., Nakauchi H., Miyoshi H. (2003). CD226 (DNAM-1) is involved in lymphocyte function-associated antigen 1 costimulatory signal for naive T cell differentiation and proliferation. J. Exp. Med..

[20] Du X., de Almeida P., Manieri N., de Almeida Nagata D., Wu T.D., Harden Bowles K., Arumugam V., Schartner J., Cubas R., Mittman S. (2018). CD226 regulates natural killer cell antitumor responses via phosphorylation-mediated inactivation of transcription factor FOXO1. Proc. Natl. Acad. Sci. USA.

[21] Deng Y., Kerdiles Y., Chu J., Yuan S., Wang Y., Chen X., Mao H., Zhang L., Zhang J., Hughes T. (2015). Transcription factor Foxo1 is a negative regulator of natural killer cell maturation and function. Immunity.

[22] Yue C., Gao S., Li S., Xing Z., Qian H., Hu Y., Wang W., Hua C. (2022). TIGIT as a Promising Therapeutic Target in Autoimmune Diseases. Front. Immunol..

[23] Liu S., Zhang H., Li M., Hu D., Li C., Ge B., Jin B., Fan Z. (2013). Recruitment of Grb2 and SHIP1 by the ITT-like motif of TIGIT suppresses granule polarization and cytotoxicity of NK cells. Cell Death Differ..

[24] Braun M., Aguilera A.R., Sundarrajan A., Corvino D., Stannard K., Krumeich S., Das I., Lima L.G., Meza Guzman L.G., Li K. (2020). CD155 on Tumor Cells Drives Resistance to Immunotherapy by Inducing the Degradation of the Activating Receptor CD226 in CD8(+) T Cells. Immunity.

[25] Kawashima S., Inozume T., Kawazu M., Ueno T., Nagasaki J., Tanji E., Honobe A., Ohnuma T., Kawamura T., Umeda Y. (2021). TIGIT/CD155 axis mediates resistance to immunotherapy in patients with melanoma with the inflamed tumor microenvironment. J. Immunother. Cancer.

[26] Inozume T., Yaguchi T., Furuta J., Harada K., Kawakami Y., Shimada S. (2016). Melanoma Cells Control Antimelanoma CTL Responses via Interaction between TIGIT and CD155 in the Effector Phase. J. Investig. Dermatol..

[27] Han W., Lv J., Wang M., Wu X., Sun D., Chen W., Wang Y., Zhou W., Yang Y., Bao J. (2025). Potential Roles of Serum Exosomal CD155 and its Impact on NK Cell Immunosuppression in Hepatocellular Carcinoma. Balk. Med. J..

[28] Saunders P.M., Oates C.V.L., Hare R., Burgess M., Vargas Pavia T.A., Worboys J.D., Davis D.M., Brooks A.G. (2026). CD155 density on target cells drives divergent natural killer cell responses owing to DNAM-1 loss. J. Immunol..

[29] Abels E.R., Breakefield X.O. (2016). Introduction to Extracellular Vesicles: Biogenesis, RNA Cargo Selection, Content, Release, and Uptake. Cell Mol. Neurobiol..

[30] Visan K.S., Wu L.Y., Voss S., Wuethrich A., Moller A. (2023). Status quo of Extracellular Vesicle isolation and detection methods for clinical utility. Semin. Cancer Biol..

[31] Welsh J.A., Goberdhan D.C.I., O’Driscoll L., Buzas E.I., Blenkiron C., Bussolati B., Cai H., Di Vizio D., Driedonks T.A.P., Erdbrugger U. (2024). Minimal information for studies of extracellular vesicles (MISEV2023): From basic to ad-vanced approaches. J. Extracell. Vesicles.

[32] Nie Y., Sato Y., Garner R.T., Kargl C., Wang C., Kuang S., Gilpin C.J., Gavin T.P. (2019). Skeletal muscle-derived exosomes regulate endothelial cell functions via reactive oxygen species-activated nuclear factor-kappaB signalling. Exp. Physiol..

[33] Bardi G.T., Smith M.A., Hood J.L. (2018). Melanoma exosomes promote mixed M1 and M2 macrophage polarization. Cytokine.

[34] Wen S.W., Lima L.G., Lobb R.J., Norris E.L., Hastie M.L., Krumeich S., Moller A. (2019). Breast Cancer-Derived Exosomes Reflect the Cell-of-Origin Phenotype. Proteomics.

[35] Whiteside T.L. (2016). Tumor-Derived Exosomes and Their Role in Tumor-Induced Immune Suppression. Vaccines.

[36] Mashouri L., Yousefi H., Aref A.R., Ahadi A.M., Molaei F., Alahari S.K. (2019). Exosomes: Composition, biogenesis, and mechanisms in cancer metastasis and drug resistance. Mol. Cancer.

[37] Pan Y., Wang Y., Wang Y., Xu S., Jiang F., Han Y., Hu M., Liu Z. (2023). Platelet-derived microvesicles (PMVs) in cancer progression and clinical applications. Clin. Transl. Oncol..

[38] Menck K., Sivaloganathan S., Bleckmann A., Binder C. (2020). Microvesicles in Cancer: Small Size, Large Potential. Int. J. Mol. Sci..

[39] Lobb R.J., van Amerongen R., Wiegmans A., Ham S., Larsen J.E., Moller A. (2017). Exosomes derived from mesenchymal non-small cell lung cancer cells promote chemoresistance. Int. J. Cancer.

[40] Peinado H., Aleckovic M., Lavotshkin S., Matei I., Costa-Silva B., Moreno-Bueno G., Hergueta-Redondo M., Williams C., Garcia-Santos G., Ghajar C. (2012). Melanoma exosomes educate bone marrow progenitor cells toward a pro-metastatic phenotype through MET. Nat. Med..

[41] Kim D.H., Kim H., Choi Y.J., Kim S.Y., Lee J.E., Sung K.J., Sung Y.H., Pack C.G., Jung M.K., Han B. (2019). Exosomal PD-L1 promotes tumor growth through immune escape in non-small cell lung cancer. Exp. Mol. Med..

[42] Lima L.G., Ham S., Shin H., Chai E.P.Z., Lek E.S.H., Lobb R.J., Muller A.F., Mathivanan S., Yeo B., Choi Y. (2021). Tumor microenvironmental cytokines bound to cancer exosomes determine uptake by cytokine receptor-expressing cells and biodistribution. Nat. Commun..

[43] Zheng Q., Wang B., Gao J., Xin N., Wang W., Song X., Shao Y., Zhao C. (2018). CD155 knockdown promotes apoptosis via AKT/Bcl-2/Bax in colon cancer cells. J. Cell. Mol. Med..

[44] Zheng Q., Gao J., Yin P., Wang W., Wang B., Li Y., Zhao C. (2020). CD155 contributes to the mesenchymal phenotype of triple-negative breast cancer. Cancer Sci..

[45] Briukhovetska D., Suarez-Gosalvez J., Voigt C., Markota A., Giannou A.D., Schubel M., Jobst J., Zhang T., Dorr J., Markl F. (2023). T cell-derived interleukin-22 drives the expression of CD155 by cancer cells to suppress NK cell function and promote metastasis. Immunity.

[46] Guetter S., Konig C., Koerkel-Qu H., Markiewicz A., Scheitler S., Katzer M., Berneburg M., Renner P., Cucuruz B., Guttenberger L. (2025). MCSP(+) metastasis founder cells activate immunosuppression early in human melanoma metastatic colonization. Nat. Cancer.

[47] Baury B., Masson D., McDermott B.M., Jarry A., Blottiere H.M., Blanchardie P., Laboisse C.L., Lustenberger P., Racaniello V.R., Denis M.G. (2003). Identification of secreted CD155 isoforms. Biochem. Biophys. Res. Commun..

[48] Koike S., Horie H., Ise I., Okitsu A., Yoshida M., Iizuka N., Takeuchi K., Takegami T., Nomoto A. (1990). The poliovirus receptor protein is produced both as membrane-bound and secreted forms. EMBO J..

[49] Dozio V., Sanchez J.C. (2017). Characterisation of extracellular vesicle-subsets derived from brain endothelial cells and analysis of their protein cargo modulation after TNF exposure. J. Extracell. Vesicles.

[50] Kowal J., Arras G., Colombo M., Jouve M., Morath J.P., Primdal-Bengtson B., Dingli F., Loew D., Tkach M., Thery C. (2016). Proteomic comparison defines novel markers to characterize heterogeneous populations of extracellular vesicle subtypes. Proc. Natl. Acad. Sci. USA.

[51] Crescitelli R., Lasser C., Jang S.C., Cvjetkovic A., Malmhall C., Karimi N., Hoog J.L., Johansson I., Fuchs J., Thorsell A. (2020). Subpopulations of extracellular vesicles from human metastatic melanoma tissue identified by quantitative proteomics after optimized isolation. J. Extracell. Vesicles.

[52] Zhou Q., Yan Y., Li Y., Fu H., Lu D., Li Z., Wang Y., Wang J., Zhu H., Ren J. (2022). Tumor-derived extracellular vesicles in melanoma immune response and immunotherapy. Biomed. Pharmacother..

[53] Dieterich L.C. (2022). Mechanisms of extracellular vesicle-mediated immune evasion in melanoma. Front. Immunol..

[54] Kluszczynska K., Czyz M. (2023). Extracellular Vesicles-Based Cell-Cell Communication in Melanoma: New Perspectives in Diagnostics and Therapy. Int. J. Mol. Sci..

[55] Tucci M., Mannavola F., Passarelli A., Stucci L.S., Cives M., Silvestris F. (2018). Exosomes in melanoma: A role in tumor progression, metastasis and impaired immune system activity. Oncotarget.

[56] Sharma P., Diergaarde B., Ferrone S., Kirkwood J.M., Whiteside T.L. (2020). Melanoma cell-derived exosomes in plasma of melanoma patients suppress functions of immune effector cells. Sci. Rep..

[57] Tarazona R., Duran E., Solana R. (2015). Natural Killer Cell Recognition of Melanoma: New Clues for a More Effective Immunotherapy. Front. Immunol..

[58] Morgado S., Sanchez-Correa B., Casado J.G., Duran E., Gayoso I., Labella F., Solana R., Tarazona R. (2011). NK cell recognition and killing of melanoma cells is controlled by multiple activating receptor-ligand interactions. J. Innate Immun..

[59] Rahimi A., Malakoutikhah Z., Rahimmanesh I., Ferns G.A., Nedaeinia R., Ishaghi S.M.M., Dana N., Haghjooy Javanmard S. (2023). The nexus of natural killer cells and melanoma tumor microenvironment: Crosstalk, chemotherapeutic potential, and innovative NK cell-based therapeutic strategies. Cancer Cell Int..

[60] Chauvin J.M., Zarour H.M. (2020). TIGIT in cancer immunotherapy. J. Immunother. Cancer.

[61] Zeng T., Cao Y., Jin T., Tian Y., Dai C., Xu F. (2021). The CD112R/CD112 axis: A breakthrough in cancer immunotherapy. J. Exp. Clin. Cancer Res..

[62] Johnston R.J., Comps-Agrar L., Hackney J., Yu X., Huseni M., Yang Y., Park S., Javinal V., Chiu H., Irving B. (2014). The immunoreceptor TIGIT regulates antitumor and antiviral CD8(+) T cell effector function. Cancer Cell.

[63] Yu X., Harden K., Gonzalez L.C., Francesco M., Chiang E., Irving B., Tom I., Ivelja S., Refino C.J., Clark H. (2009). The surface protein TIGIT suppresses T cell activation by promoting the generation of mature immunoregulatory dendritic cells. Nat. Immunol..

[64] Anderson A.C., Joller N., Kuchroo V.K. (2016). Lag-3, Tim-3, and TIGIT: Co-inhibitory Receptors with Specialized Functions in Immune Regulation. Immunity.

[65] Hasan M.F., Campbell A.R., Croom-Perez T.J., Oyer J.L., Dieffenthaller T.A., Robles-Carrillo L.D., Cash C.A., Eloriaga J.E., Kumar S., Andersen B.W. (2023). Knockout of the inhibitory receptor TIGIT enhances the antitumor response of ex vivo expanded NK cells and prevents fratricide with therapeutic Fc-active TIGIT antibodies. J. Immunother. Cancer.

[66] Fang C., Li G., Han M., Wang Y., Yu W., Hu D., Luo J., Wang L., Fan W., Wang L. (2026). Base Editing of TIGIT Reprograms CD155 Signaling in Natural Killer Cells to Enhance Cancer Immunotherapy Efficacy. Cancer Res..

[67] Stanietsky N., Rovis T.L., Glasner A., Seidel E., Tsukerman P., Yamin R., Enk J., Jonjic S., Mandelboim O. (2013). Mouse TIGIT inhibits NK-cell cytotoxicity upon interaction with PVR. Eur. J. Immunol..

[68] Okumura G., Iguchi-Manaka A., Murata R., Yamashita-Kanemaru Y., Shibuya A., Shibuya K. (2020). Tumor-derived soluble CD155 inhibits DNAM-1-mediated antitumor activity of natural killer cells. J. Exp. Med..

[69] Iguchi-Manaka A., Okumura G., Kojima H., Cho Y., Hirochika R., Bando H., Sato T., Yoshikawa H., Hara H., Shibuya A. (2016). Increased Soluble CD155 in the Serum of Cancer Patients. PLoS ONE.

[70] Alegre E., Howangyin K.Y., Favier B., Baudhuin J., Lesport E., Daouya M., Gonzalez A., Carosella E.D., Lemaoult J. (2010). Membrane redistributions through multi-intercellular exchanges and serial trogocytosis. Cell Res..

[71] Caumartin J., Favier B., Daouya M., Guillard C., Moreau P., Carosella E.D., LeMaoult J. (2007). Trogocytosis-based generation of suppressive NK cells. EMBO J..

[72] Mirjacic Martinovic K.M., Babovic N., Dzodic R.R., Jurisic V.B., Tanic N.T., Konjevic G.M. (2014). Decreased expression of NKG2D, NKp46, DNAM-1 receptors, and intracellular perforin and STAT-1 effector molecules in NK cells and their dim and bright subsets in metastatic melanoma patients. Melanoma Res..

[73] Molfetta R., Zitti B., Lecce M., Milito N.D., Stabile H., Fionda C., Cippitelli M., Gismondi A., Santoni A., Paolini R. (2020). CD155: A Multi-Functional Molecule in Tumor Progression. Int. J. Mol. Sci..

[74] Paolini R., Molfetta R. (2023). CD155 and Its Receptors as Targets for Cancer Therapy. Int. J. Mol. Sci..

[75] Chan C.J., Andrews D.M., McLaughlin N.M., Yagita H., Gilfillan S., Colonna M., Smyth M.J. (2010). DNAM-1/CD155 interactions promote cytokine and NK cell-mediated suppression of poorly immunogenic melanoma metastases. J. Immunol..

[76] Carlsten M., Norell H., Bryceson Y.T., Poschke I., Schedvins K., Ljunggren H.G., Kiessling R., Malmberg K.J. (2009). Primary human tumor cells expressing CD155 impair tumor targeting by down-regulating DNAM-1 on NK cells. J. Immunol..

[77] Shevtsov M., Pitkin E., Ischenko A., Stangl S., Khachatryan W., Galibin O., Edmond S., Lobinger D., Multhoff G. (2019). Ex vivo Hsp70-Activated NK Cells in Combination with PD-1 Inhibition Significantly Increase Overall Survival in Preclinical Models of Glioblastoma and Lung Cancer. Front. Immunol..

[78] Bald T., Krummel M.F., Smyth M.J., Barry K.C. (2020). The NK cell-cancer cycle: Advances and new challenges in NK cell-based immunotherapies. Nat. Immunol..

[79] Jella K.K., Nasti T.H., Li Z., Lawson D.H., Switchenko J.M., Ahmed R., Dynan W.S., Khan M.K. (2020). Exosome-Containing Preparations From Postirradiated Mouse Melanoma Cells Delay Melanoma Growth In Vivo by a Natural Killer Cell-Dependent Mechanism. Int. J. Radiat. Oncol. Biol. Phys..

[80] Xiong T., Wang G., Yu P., Li Z., Li D., Zhang J., Lu S., Yang R., Lian X., Mi J. (2025). CAR-T cells targeting CD155 reduce tumor burden in preclinical models of leukemia and solid tumors. J. Clin. Investig..

[81] Abe A., Fukui H., Fujii S., Kono T., Mukawa K., Yoshitake N., Sekikawa A., Ichikawa K., Tomita S., Yamagishi H. (2009). Role of Necl-5 in the pathophysiology of colorectal lesions induced by dimethylhydrazine and/or dextran sodium sulphate. J. Pathol..

[82] Sceneay J., Chow M.T., Chen A., Halse H.M., Wong C.S., Andrews D.M., Sloan E.K., Parker B.S., Bowtell D.D., Smyth M.J. (2012). Primary tumor hypoxia recruits CD11b+/Ly6Cmed/Ly6G+ immune suppressor cells and compromises NK cell cytotoxicity in the premetastatic niche. Cancer Res..

[83] Kim S.H., Kim M.J., Lim M., Kim J., Kim H., Yun C.K., Yoo Y.J., Lee Y., Min K., Choi Y.S. (2023). Enhancement of the Anticancer Ability of Natural Killer Cells through Allogeneic Mitochondrial Transfer. Cancers.

[84] Lobb R.J., Becker M., Wen S.W., Wong C.S., Wiegmans A.P., Leimgruber A., Moller A. (2015). Optimized exosome isolation protocol for cell culture supernatant and human plasma. J. Extracell. Vesicles.

[85] Visan K.S., Lobb R.J., Ham S., Lima L.G., Palma C., Edna C.P.Z., Wu L.Y., Gowda H., Datta K.K., Hartel G. (2022). Comparative analysis of tangential flow filtration and ultracentrifugation, both combined with subsequent size exclusion chromatography, for the isolation of small extracellular vesicles. J. Extracell. Vesicles.

[86] Wen S.W., Sceneay J., Lima L.G., Wong C.S., Becker M., Krumeich S., Lobb R.J., Castillo V., Wong K.N., Ellis S. (2016). The Biodistribution and Immune Suppressive Effects of Breast Cancer-Derived Exosomes. Cancer Res..

