# Supplementary material for: Intercellular Transfer of CD155 Mediated by Melanoma Extracellular Vesicles Downregulates DNAM-1 Expression on NK Cells
Source: Int J Mol Sci. 2026 Sep 29;27(19):8699. doi: 10.3390/ijms27198699 (PMC13664779; doi:10.3390/ijms27198699)
Supplement: Supplementary file 1 [file ijms-27-08699-s001.zip › ijms-4516095-supplementary.pdf]

Additional information

Intercellular transfer of CD155 mediated by melanoma extracellular vesicles downregulates DNAM-1 expression on NK cells

Li-Ying Wu [1,2,3,4], Luize Goncalves Lima [1,3,4,*], Sunyoung Ham [3,4], Mina Lim [5], Su-Ho Park [3,4], Yue Su [3,4], Xuanxuan Li [3,4], Edna Pei Zhi Chai [1], Matthias Braun [6], Yong-Soo Choi [7,8], Tobias Bald [9] and Andreas Möller [1,2,3,4,*]

Supplementary material includes:

- Supplementary Figures and Legends 1-9
- Raw data of western blot.

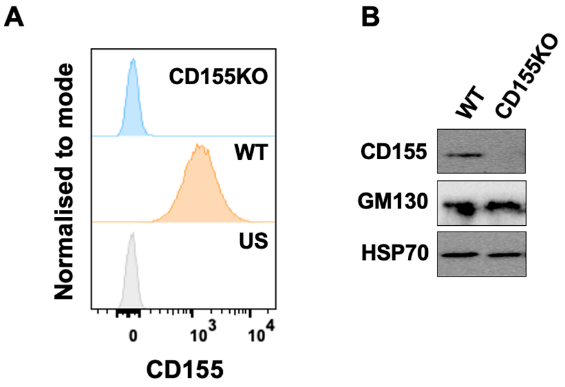

Figure S1. CD155 was not detected in B16F10-CD155KO cells. (**A**) CD155 expression on B16F10-WT and B16F10-CD155KO cells was assessed by flow cytometry. Similar results were obtained from at least three biologically independent cell preparations. (**B**) Representative immunoblots of CD155 (100 kDa), the cis-Golgi marker GM130 (112 kDa), and HSP70 (70 kDa) in cell lysates from B16F10-WT and B16F10-CD155KO cells. Similar results were obtained from at least three biologically independent cell preparations. Source data are provided as a Source Data file.

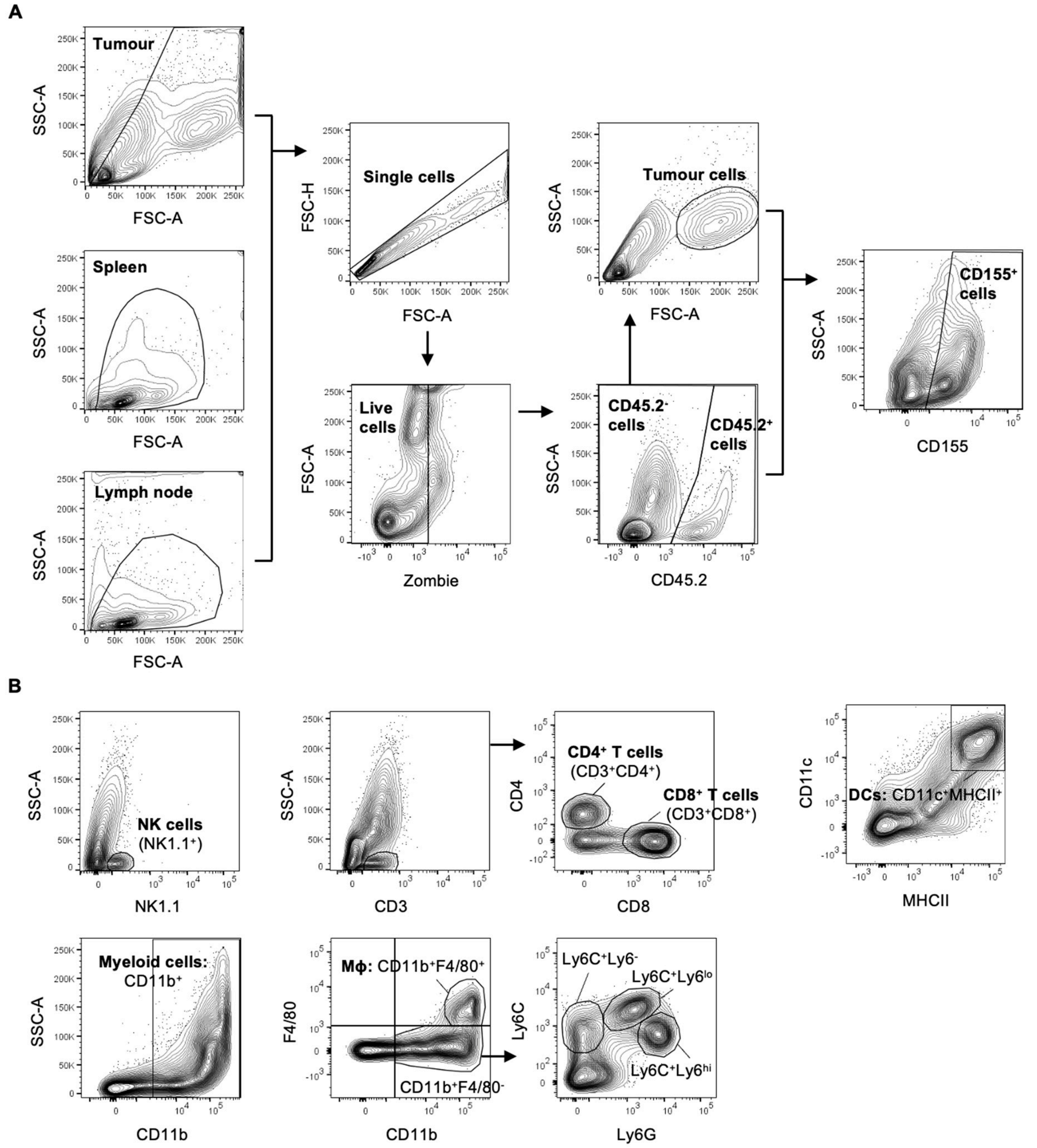

Figure S2. Gating strategy for flow cytometry analysis. (**A**) Single-cell suspensions from tumour, spleen, and lymph node tissues were analysed by flow cytometry after gating on live single cells based on FSC and SSC parameters. Zombie Yellow or Zombie Aqua was used as a viability dye. Leucocytes were selected as CD45.2^{+} cells. In tumour tissues, tumour cells were further selected as CD45.2^{-}FSC^{hi} cells. The frequency of the CD155^{+} population was assessed within both the leucocyte and tumour-cell populations. (**B**) Distinct CD45.2^{+} immune-cell subsets were selected as follows: NK1.1^{+} for NK cells; CD3^{+}CD4^{+} or CD3^{+}CD8^{+} for T cells; CD11c^{+}MHCII^{+} for dendritic cells (DCs); CD11b^{+} for whole myeloid cell population; CD11b^{+}F4/80^{+}, for macrophages (Mϕ). Subsets of myeloid cells were further selected from CD11b^{+}F4/80^{-} cells as: CD11b^{+}Ly6C^{+}Ly6G^{-}, CD11b^{+}Ly6C^{+}Ly6G^{lo} or CD11b^{+}Ly6C^{+}Ly6G^{hi}. Frequency of CD155^{+} population was also assessed within each CD45.2^{+} immune cell subset. Representative images are shown.

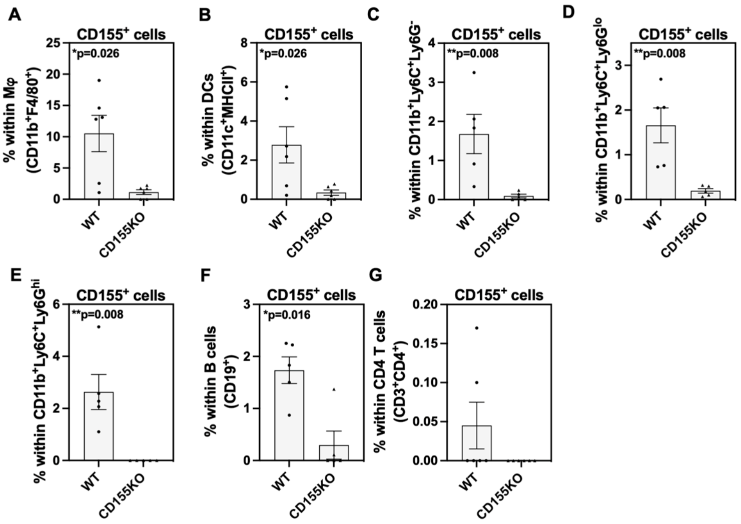

Figure S3. CD155 was transferred from B16F10-WT melanoma cells to CD155-/- immune-cell subsets. Frequency of $CD155^+$ cells within distinct $CD45^+$ immune-cell subsets of WT and CD155KO tumours, including macrophage (M**φ**; n = 5 mice per group); **A**), dendritic cells (DCs; n = 6 mice per group)**; B**), myeloid-derived suppressor cells subsets (n = 5 mice per group)**; C-E**), B cells (n = 5 mice per group)**; F**) and CD4 T cells (n = 6 mice per group). Data are presented as mean ± SEM. **p* < 0.05 and ***p* <0.01, as analysed by the two-tailed Mann–Whitney U test.

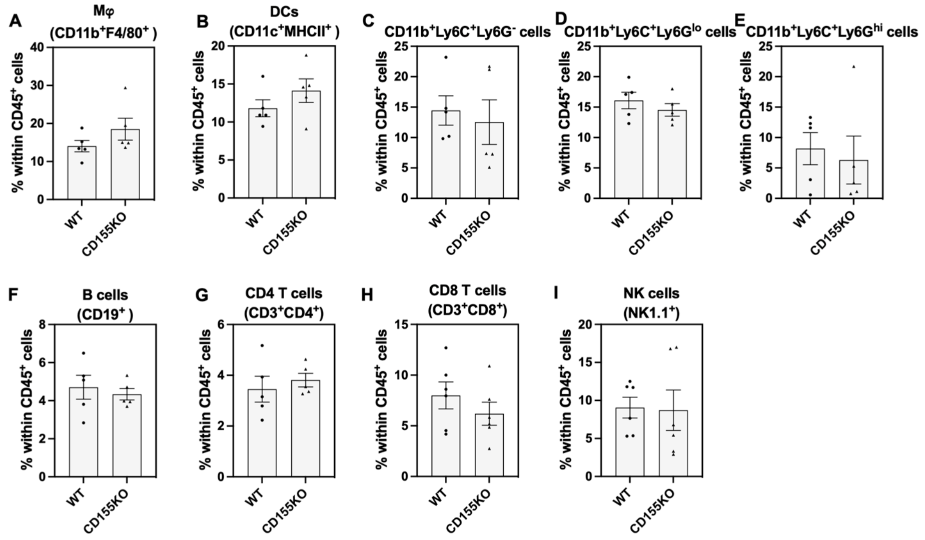

Figure S4. Frequency of immune-cell subsets within the primary tumour. Cells were isolated from tumours of B16F10-WT (WT) and B16F10-CD155KO (CD155KO) melanoma-bearing $CD155^{-/-}$ mice. Frequencies of different $CD45^+$ immune-cell subsets within WT and CD155KO tumours are shown in (A-G, n = 5 mice per group) and (H-I, n= 6 mice per group). (**A**) Macrophage (M**φ**), (**B**) dendritic cells (DCs), (**C-E**) distinct subsets of myeloid-derived suppressor cells, (**F**) B cells, (**G**) CD4 T cells, (**H**) CD8 T cells, and (**I**) NK cells are shown. Data are presented as mean ± SEM.

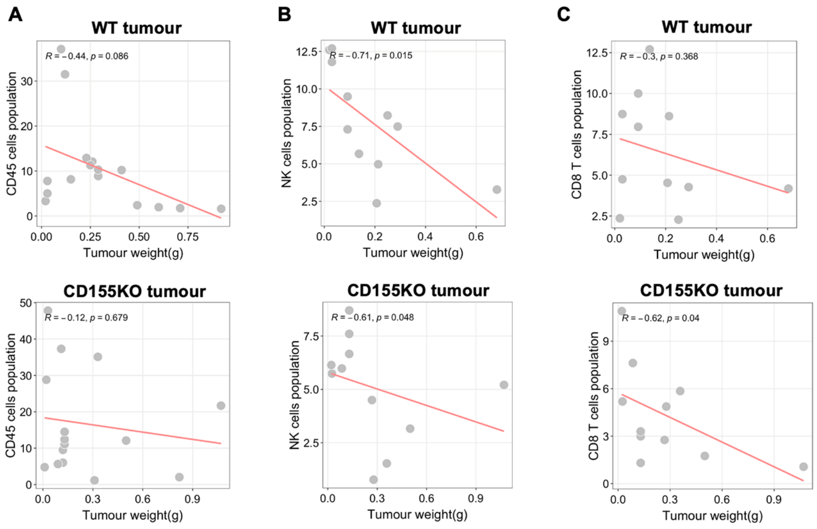

Figure S5. Frequencies of immune-cell subsets within the tumour microenvironment, particularly NK cells, are inversely correlated with tumour size. Cells were isolated from tumours of B16F10-WT (WT) and B16F10-CD155KO (CD155KO) melanoma-bearing CD155$^{-/-}$ mice. The percentage of distinct immune-cell subsets within live cells in individual WT and CD155KO tumours were plotted against the corresponding tumour weights to assess their correlation. (**A**) CD45^{+} cells, (**B**) NK cells (NK1.1^{+}), and (**C**) CD8 T cells (CD3^{+}CD8^{+}) are shown. Data were analysed using the Spearman correlation method, and the plots were created in RStudio.

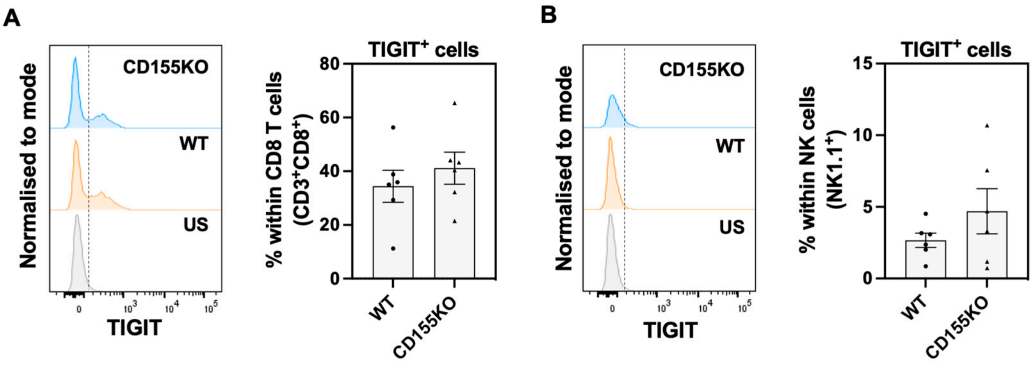

Figure S6. Analysis of TIGIT expression reveals no difference between CD8 T cells and NK cells infiltrating B16F10-WT tumours compared with B16F10-CD155KO tumours. Single-cell suspensions were prepared from tumours of B16F10-WT (WT) and B16F10-CD155KO (CD155KO) melanoma-bearing CD155$^{-/-}$ mice. (**A**) Histograms showing TIGIT expression (left) and the quantified frequency of TIGIT^{+} cells (right) within the CD8 T-cell population. (**B**) Histograms showing TIGIT expression (left) and the quantified frequency of TIGIT^{+} cells (right) within the NK cell population. Data are presented as mean ± SEM (n = 6 mice per group). *$p < 0.05$ and **$p < 0.01$, as analysed by the two-tailed Mann–Whitney U test.

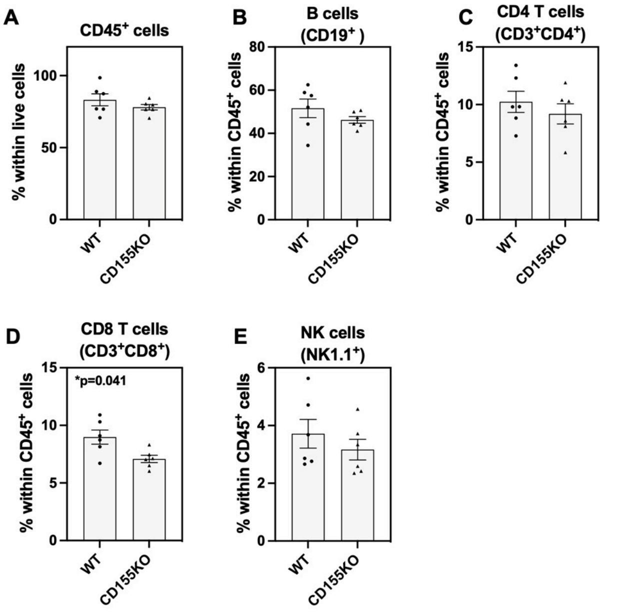

Figure S7. Immune-cell composition of spleens from B16F10-WT and B16F10-CD155KO tumour-bearing CD155$^{-/-}$ mice. Single-cell suspensions were prepared from spleens of B16F10-WT (WT) and B16F10-CD155KO (CD155KO) melanoma-bearing CD155$^{-/-}$ mice. The frequencies of CD45^{+} cells within live cells (**A**), and of distinct immune-cell subsets within the CD45^{+} population, including B cells (**B**), CD4 T cells (**C**), CD8 T cells (**D**), and NK cells (**E**), were analysed by flow cytometry (n = 6 mice per group). Data are presented as mean ± SEM. *p < 0.05, as analysed by the two-tailed Mann–Whitney U test.

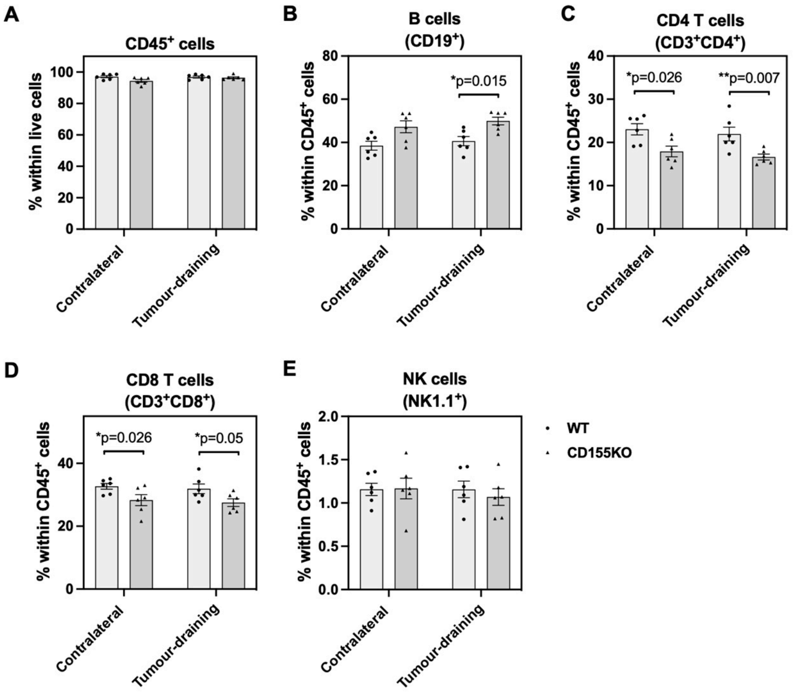

Figure S8. Immune-cell composition of lymph nodes from B16F10-WT and B16F10-CD155KO tumour-bearing CD155$^{-/-}$ mice. Single-cell suspensions were prepared from lymph nodes, including contralateral and tumour-draining lymph nodes, from B16F10-WT (WT) and B16F10-CD155KO (CD155KO) melanoma-bearing CD155$^{-/-}$ mice. The frequencies of CD45^{+} cells within live cells (**A**), as well as of distinct immune-cell subsets including B cells (**B**), CD4 T cells (**C**), CD8 T cells (**D**), NK cells (**E**), were analysed by flow cytometry (n = 6 mice per group). Data are presented as mean ± SEM. *p < 0.05, **p < 0.01 as analysed by the two-tailed Mann–Whitney U test.

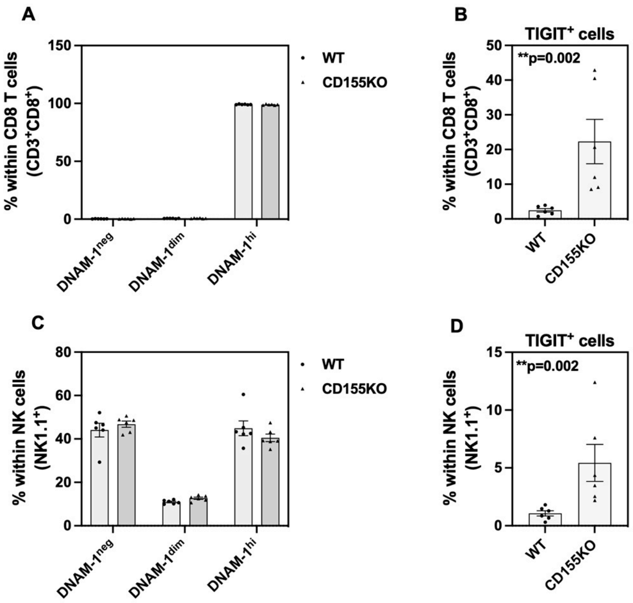

Figure S9. TIGIT expression is increased in CD8 T and NK cells present in the spleens of B16F10-CD155KO tumour-bearing CD155$^{-/-}$ mice. Single-cell suspensions were prepared from spleens of B16F10-WT (WT) and B16F10-CD155KO (CD155KO) melanoma-bearing CD155$^{-/-}$ mice. The frequencies of CD155 receptors, DNAM-1^{+} cells or TIGIT^{+} cells within distinct immune-cell subsets, including CD8 T cells (**A and B**) and NK cells (**C and D**), were analysed (n = 6 mice per group). Data are presented as mean ± SEM. **p <0.01, as analysed by the two-tailed Mann–Whitney U test.

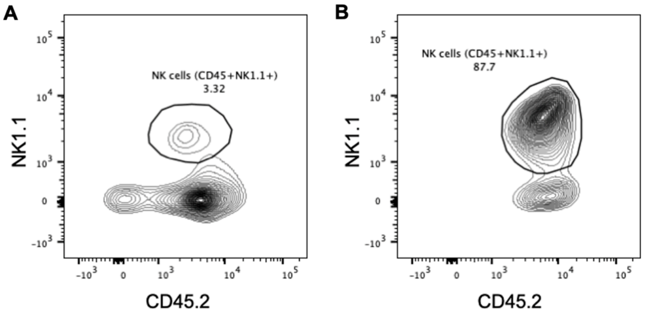

Figure S10. Primary murine NK cells isolated from the spleens of CD155$^{-/-}$ mice. Primary NK cells were isolated from whole spleens of CD155$^{-/-}$ mice by negative selection (see Material and Methods section). Representative dot plots show the percentage of CD45^{+}NK1.1^{+} cells within the total population of live single cells before (**A**) and after (**B**) the isolation procedure.

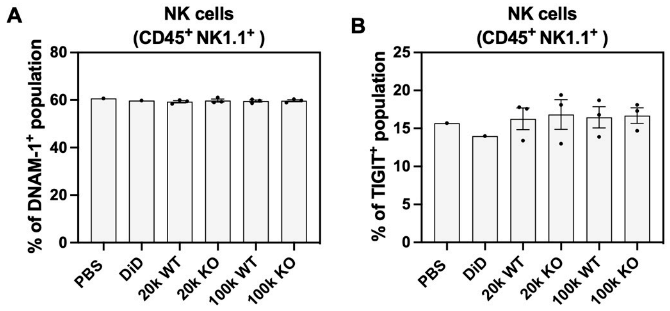

Figure S11. CD155 receptors are not increased in murine NK cells after EV incubation. Primary NK cells were isolated from whole spleens of CD155$^{-/-}$ mice by negative selection (see Material and Methods section), and cultured overnight in IL-2 containing medium. Cells were then treated with 30 ug of DiD-labelled B16F10-WT (WT) or B16F10-CD155KO (KO) 20k or 100k EVs for 24 hours and analysed by flow cytometry (n = 3 biologically independent samples per group). PBS and DiD were used as controls. (A) Frequency of DNAM-1$^+$ cells within CD45$^+$NK1.1$^+$ NK cells. (B) Frequency of TIGIT$^+$ cells within CD45$^+$NK1.1$^+$ NK cells.

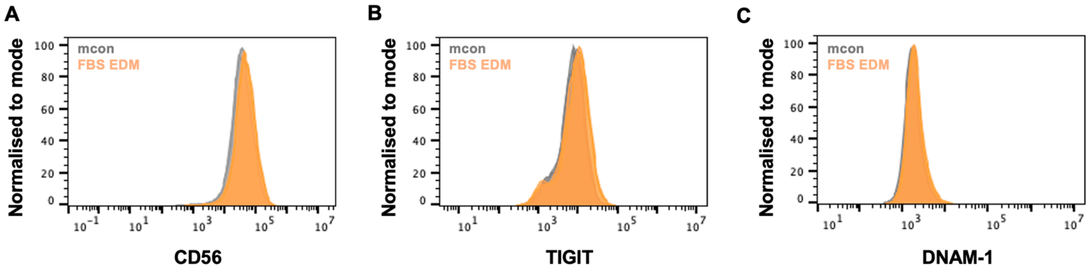

Figure S12. EDM has no effect on the expression of CD56 and CD155 receptors by human NK cells. NK-92MI human NK cells were cultured in different conditioned media, including normal culture medium (mcon; see Methods section) and EV-depleted media (FBS EDM; 12.5% EDM, 0.2 mM myo-Inositol, 0.1 mM 2-Mercaptoethanol, 0.02 mM Folic acid and Minimum Essential Medium α), and analysed by flow cytometry. (**A**) CD56 expression in live NK-92MI cells. (**B**) TIGIT expression in CD56$^+$ NK-92MI cells. (**C**) DNAM-1 expression in CD56$^+$ NK-92MI cells. Representative histograms from three biologically independent samples per group are shown.

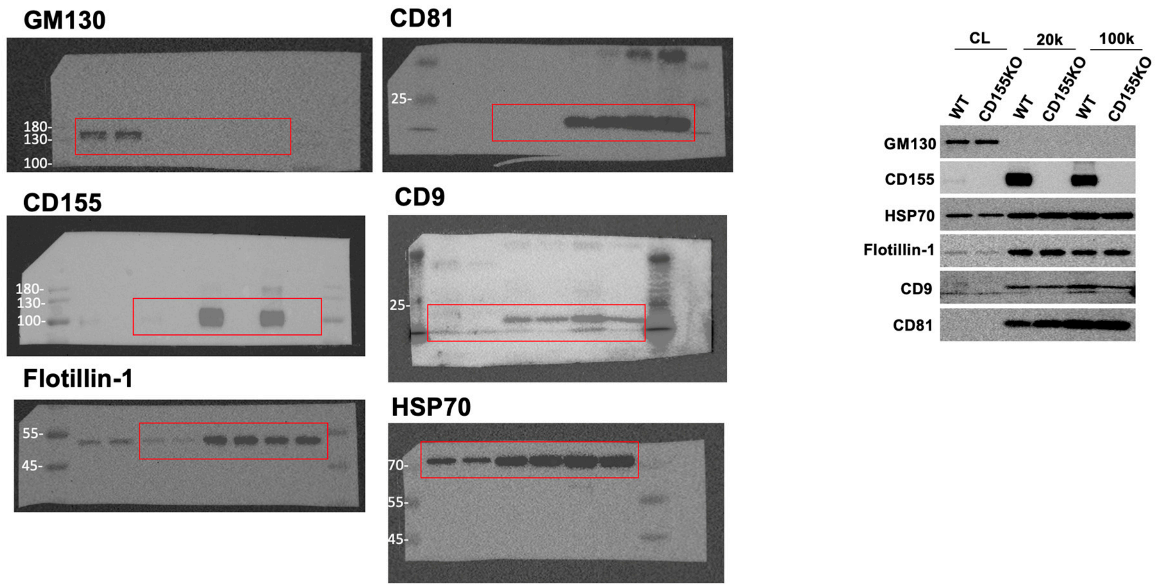

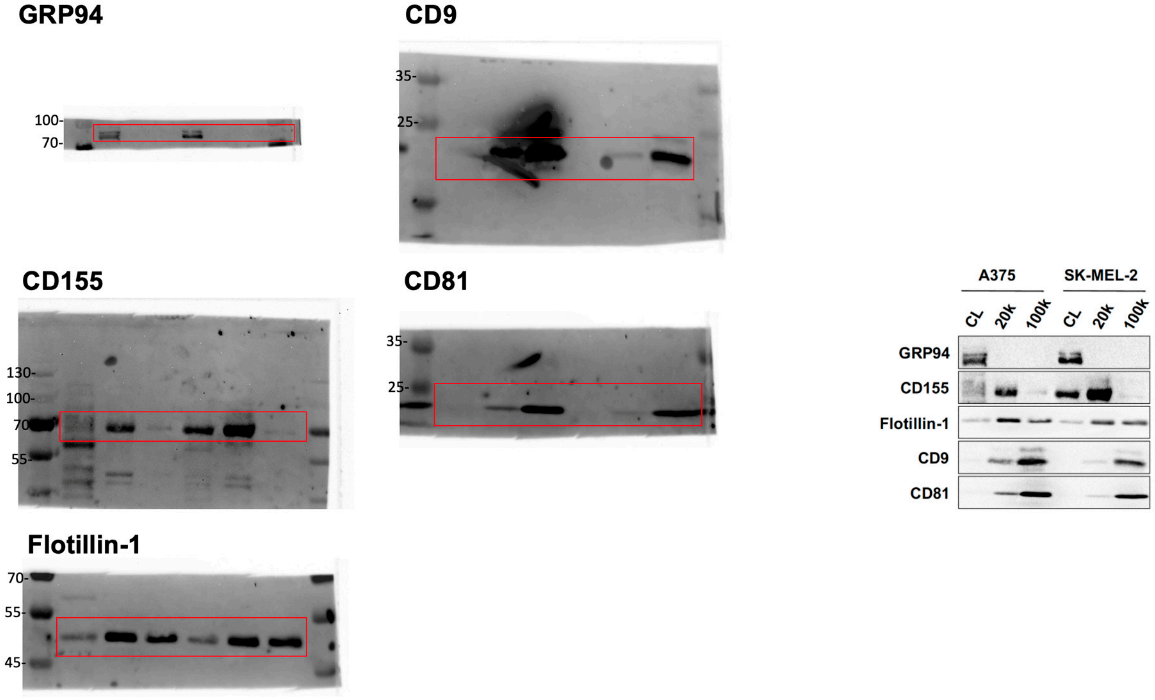

Raw data of western blot.
